# Same same, but different: exploring the enigmatic role of the pituitary adenylate cyclase-activating polypeptide (PACAP) in invertebrate physiology

**DOI:** 10.1007/s00359-024-01706-5

**Published:** 2024-06-28

**Authors:** Zsolt Pirger, Péter Urbán, Bence Gálik, Bence Kiss, Antal Tapodi, János Schmidt, Gábor K. Tóth, Joris M. Koene, György Kemenes, Dóra Reglődi, Tibor Kiss, István Fodor

**Affiliations:** 1 Ecophysiological and Environmental Toxicological Research Group, HUN-REN Balaton Limnological Research Institute, Tihany, 8237 Hungary; 2 Genomics and Bioinformatics Core Facilities, Szentágothai Research Centre, University of Pécs, Pécs, 7624 Hungary; 3 Institute of Biochemistry and Medical Chemistry, Medical School, University of Pécs, Pécs, 7624 Hungary; 4 Department of Medical Chemistry, University of Szeged, Szeged, Hungary; 5 Ecology & Evolution, Amsterdam Institute for Life and Environment, Faculty of Science, Vrije Universiteit, Amsterdam, the Netherlands; 6 Sussex Neuroscience, School of Life Sciences, University of Sussex, Brighton, BN1 9QG UK; 7 Department of Anatomy, ELKH-PTE PACAP Research Team, Centre for Neuroscience, Medical School, University of Pécs, Pécs, 7624 Hungary

**Keywords:** PACAP, Protostomes, *Lymnaea stagnalis*, Evolution, Physiology

## Abstract

Evidence has been accumulating that elements of the vertebrate pituitary adenylate cyclase-activating polypeptide (PACAP) system are missing in non-chordate genomes, which is at odds with the partial sequence-, immunohistochemical-, and physiological data in the literature. Multilevel experiments were performed on the great pond snail (*Lymnaea stagnalis*) to explore the role of PACAP in invertebrates. Screening of neuronal transcriptome and genome data did not reveal homologs to the elements of vertebrate PACAP system. Despite this, immunohistochemical investigations with an anti-human PAC_1_ receptor antibody yielded a positive signal in the neuronal elements in the heart. Although Western blotting of proteins extracted from the nervous system found a relevant band for PACAP-38, immunoprecipitation and mass spectrometric analyses revealed no corresponding peptide fragments. Similarly to the effects reported in vertebrates, PACAP-38 significantly increased cAMP synthesis in the heart and had a positive ionotropic effect on heart preparations. Moreover, it significantly modulated the effects of serotonin and acetylcholine. Homologs to members of Cluster B receptors, which have shared common evolutionary origin with the vertebrate PACAP receptors, PTHRs, and GCGRs, were identified and shown not to be expressed in the heart, which does not support a potential role in the mediation of PACAP-induced effects. Our findings support the notion that the PACAP system emerged after the protostome-deuterostome divergence. Using antibodies against vertebrate proteins is again highlighted to have little/no value in invertebrate studies. The physiological effects of vertebrate PACAP peptides in protostomes, no matter how similar they are to those in vertebrates, should be considered non-specific.

**Supplementary Information:**

The online version contains supplementary material available at 10.1007/s00359-024-01706-5.

## Introduction

The pituitary adenylate cyclase-activating polypeptide (PACAP) is a member of the Secretin neuropeptide superfamily (Supplementary Table 1) and one of the most extensively studied neuropeptides (reviewed by (Cardoso et al. 2020). In vertebrates, except teleosts, the PACAP precursor is encoded by a single gene (ADCYAP1) (Cardoso et al. 2020; Sherwood et al. 2000; Vaudry et al. 2000, 2009) and gives rise to two biologically active peptide isoforms: the predominant PACAP-38 and the shorter PACAP-27 (Arimura and Shioda 1995; Miyata et al. 1989, 1990; Sherwood et al. 2000; Vaudry et al. 2000, 2009). In vertebrates, the active peptides primarily bind to the PAC_1_ receptor, which belongs to the B1 GPCR family, but can also activate VPAC_1_ and VPAC_2_ receptors, which also bind the closest homologous peptide (vasoactive intestinal peptide) with equal affinity (Moody et al. 2021; Vaudry et al. 2009). In all vertebrate taxa, when PACAP binds to its receptor(s), it activates a series of intracellular signaling pathway, mainly the production of cAMP or the mobilization of calcium ions (Cardoso et al. 2020).

A large body of literature has attempted to provide evidence of a homolog of the vertebrate PACAP system in non-bilaterians and protostomes for more than 20 years (reviewed by (Cardoso et al. 2020). A common line of evidence is that partial cDNA sequences encoding a PACAP-like peptide, highly similar to the human PACAP (> 87%) or identical to the teleost PACAP, have been isolated in a few cnidarian, cephalopod, and arthropod species (Cardoso et al. 2020; Kiss and Pirger 2013; Lugo et al. 2013; Pirger et al. 2016). Another line of evidence is that cells in protostome tissues can be immunohistochemically stained with antibodies raised against vertebrate PACAP peptides and their receptors (Boros et al. 2008, 2010; Hernadi et al. 2008; Krajcs et al. 2015; Molnar et al. 2006, 2008; Pirger et al. 2008, 2010b; Reglodi et al. 2000; Somogyi et al. 2009; Varhalmi et al. 2008; Zhong and Pena 1995). The last one is that vertebrate PACAP peptides seem to be able to cause physiological changes in protostomes similar to those in vertebrates (Bhattacharya et al. 2004; Hernadi et al. 2008; Krajcs et al. 2015; Lugo et al. 2013; Maasz et al. 2017; Pirger et al. 2008, 2010a, b, 2014; Zhong 1995; Zhong and Pena 1995). However, it is remarkable that initial major data-mining of public non-bilaterian, protostome, and early-deuterostome genome and transcriptome data could not identify sequences that are homologous to genes encoding vertebrate PACAP peptides and their receptors (Cardoso et al. 2007, 2010; Mirabeau and Joly 2013). Moreover, taking advantage of the extensively increasing invertebrate sequence data, a recent elegant paper made comprehensive sequence searches in numerous invertebrate species (> 3000 transcriptome and genome data) and revisited the evolution of the PACAP system (Cardoso et al. 2020). The major conclusions of this paper by Cardoso and his co-workers were that (1) “elements of the vertebrate PACAP system are absent from protozoans, non-bilaterians, and protostomes” and (2) “PACAP and its receptors appeared in vertebrate genomes and probably shared a common ancestral origin with the cephalochordate PACAP/glucagon-like system” (Cardoso et al. 2020). These facts are clearly at odds, however, with the partial sequence-, immunohistochemical-, and physiological evidence (detailed in Supplementary Table 2) in the literature mentioned above.

The aim of the present study was to further trace the origins and functions of vertebrate PACAP system and to move the debate in protostomes forward by investigating the great pond snail (*Lymnaea stagnalis*). Since this snail has been a widely used model organism in neuroscience for decades (reviewed by (Benjamin 2008; Benjamin et al. 2021; Fodor et al. 2020, 2021; Kemenes and Benjamin 2009; Rivi et al. 2020, 2021), as well as a subject of protostome PACAP research for many years (Kiss and Pirger 2013; Maasz et al. 2017; Pirger et al. 2010a, b, 2014, 2016), it is highly suitable for such investigations. To accomplish our aim, we first sequenced the whole neural transcriptome of *L. stagnalis* and screened it for homologs to the elements of the vertebrate PACAP system. Also, we performed immunohistochemistry (IHC) using antisera generated against the human PACAP-38 peptide and human PAC_1_ receptor to investigate whether these yielded positive signals in the heart. We also used Polyacrylamide Gel (PAGE) separation of central nervous system (CNS) and heart extracts, as well as hemolymph, followed by Western blotting (WB), immunoprecipitation, and mass spectrometry (MS) analyses to try to at least partially characterize polypeptide(s) that previously showed cross-reactivity with the anti-human PACAP-38 antibody in the CNS. In addition, we performed in vitro pharmacological experiments on heart preparations to investigate whether PACAP-38 has any direct or modulatory effect on this organ. Our findings support the idea that PACAP and its receptors are absent in non-chordate animals and emerged after the protostome-deuterostome divergence.

## Materials and methods

### Experimental animals

For this study, 5-month-old (adult) *L. stagnalis* specimens were randomly obtained from our laboratory-bred stocks (Balaton Limnological Research Institute). Snails were maintained in large holding tanks (density: 100 individuals/tank) containing 10 L oxygenated artificial snail water with low copper content at 20 °C (± 1 °C) on a 12 h light:12 h dark regime. Specimens were fed on lettuce ad libitum three times a week.

### Nucleotide sequencing and bioinformatic analysis

The whole CNS was dissected from the snails (*n* = 10) and homogenized using a TissueLyser LT (QIAGEN) in TRI reagent (#93,289, Merck). RNA was isolated with Direct-zol™ RNA MiniPrep (#R2050, Zymo Research) following manufacturer’s instructions. The RNA was quantified by a Qubit 3.0 device using BR RNA Kit (#Q10211, Thermo Fisher Scientific) and the quality was checked on Agilent Bioanalyzer 2100 using RNA 6000 Nano Kit (#5067 − 1511, Agilent).

Nanopore- and Illumina-sequencing were used for identification of any homolog in the neuronal transcriptome of *L. stagnalis* to the chordate and vertebrate PACAP prepropeptides, active peptides, and their receptors. In the case of Nanopore-seq, the library was prepared using the cDNA-PCR Kit (#SQK-PCS108, Oxford Nanopore Technologies) according to the manufacturer’s description. The sample was sequenced on a MinION device with R9.4.1 flowcells (#FLO-MIN106, Oxford Nanopore Technologies). Real-time base calling was performed using Guppy v3.2.2 software. Adapters were trimmed with Porechop v9.0 (Wick et al. 2018) and sequences with internal adapters (chimera reads) were also split with Porechop. Filtering on quality and read length was performed with Nanofilt (De Coster et al. 2018). Read assembly, manual correction, and consensus sequence extraction were made using the CLC Genomics Workbench v12.0.3 software *de novo* pipeline (QIAGEN). In the case of Illumina-seq, the library was prepared using NEBNext Ultra II Directional RNA Library Prep Kit for Illumina (#E7765L, New England Biolabs). Briefly, mRNA was isolated from 400 ng total RNA using NEBNext Poly(A) mRNA Magnetic Isolation Module (#E7490L, New England Biolabs). Thereafter, the mRNA was fragmented, end prepped, and adapter-ligated. Finally, the library was amplified according to the manufacturer’s instructions. The quality of the libraries was checked on 4200 TapeSation System using D1000 Screen Tape (#5067–5582, Agilent), the quantity was measured on Qubit 3.0. Illumina sequencing was performed on the Illumina NovaSeq6000 instrument with 2 × 151 run configuration. Read quality was visualized using FastQC (Andrews 2010), and then analyzed using rCorrector (Song and Florea 2015), which employs a k-mer based method to correct random sequencing errors in Illumina RNA-seq reads. Reads were assembled using the Trinity *de novo* assembly program (Grabherr et al. 2011).

The publication of the neuronal transcriptome assembly and transcriptomic data (e.g., BUSCO, GO, and KEGG analyses) is in preparation - since the neuronal transcriptome is not the main topic of this manuscript and was only partly used to this study, the assembly will be published and publicly available in another paper. Following the homolog searching approach of a paper by Cardoso and his co-workers (Cardoso et al. 2020), we used a broad group of relevant sequences (mammal, bird, amphibian, fish [agnathan, teleost, non-teleost]), and cephalochordate) as search queries (Supplementary Table 3; Supplementary Table 4). As an additional investigation, homolog searchers for other members of the Secretin superfamily were also carried out (Supplementary Table 5). As a supplementary investigation, we also made the homolog searching in (1) the public *L. stagnalis* CNS transcriptome shotgun assembly (sequence read archive: #DRR002012; (Sadamoto et al. 2012) and (2) the preliminary *L. stagnalis* reference genome data to which we have access as part of the genome consortium (genome publication in preparation).

### Sequence analysis, phylogenetics, and expression of cluster B receptors

The conserved domain search was performed with the NCBI CDD/SPARCLE (Lu et al. 2020; Marchler-Bauer et al. 2017) to check if the key regions were present in the deduced Cluster B protein sequences. For 3D structure prediction, the computational modelling was made with AlphaFold2, a protein structure predicting algorithm (Jumper et al. 2021). The amino acid sequences were run through the GoogleColab platform (Bisong 2019) followed by a further examination with PyMOL (PyMOL 2020) and Swiss-PdbViewer 4.1.0 (Guex and Peitsch 1997).

The multiple sequence alignment used to generate the maximum likelihood tree consisted of the two newly identified *L. stagnalis* sequences and further 28 amino acid sequences obtained from the previous phylogenetic analysis of Cardoso and his co-workers (Cardoso et al. 2020). The alignment was performed using the ClustalW function with BLOSUM62 substitution matrix in Molecular Evolutionary Genetics Analysis v7 software30 (Kumar et al. 2016). The fitting model was LG with gamma-distributed rates. Bootstrapping support for the tree was conducted with 1000 bootstrap replicates, the bootstrap values (%) are indicated at each branch point. All positions containing gaps and missing data were eliminated. There was a total of 236 positions in the final dataset.

The CNS and heart were dissected from the animals (*n* = 5). Total RNA from the pooled tissues was isolated as presented above. To ensure that the samples are free from genomic DNA contamination, an additional DNase treatment was performed after the RNA preparation using TURBO DNA-free™ Kit (#AM1907, Thermo Fisher Scientific) following manufacturer’s instructions. The RevertAid H Minus First Strand cDNA Synthesis Kit (#K1631, Thermo Fisher Scientific) was used for reverse transcription (RT), applying random hexamer primers and 300 ng total RNA. PCR primers were designed for the identified *L. stagnalis* Cluster B sequences with the SnapGene® Viewer software (GSL Biotech, Chicago, IL, version 4.1.7). The applied primer set for the Cluster B sequence 1 was as follows: 5′ – CTA TTC CTG GCC TTC GTC CTA CG– 3′ (forward) and 5′ – AGA GGA CAC GGA CGA TGT TGA T – 3′ (reverse). The applied primer set for the Cluster B sequence 2 was as follows: 5′ – GAA CAG GTG GCT CGA ATA ACG AC– 3′ (forward) and 5′ – ATG GTG TTT CTT GAG CAG TGC AAC – 3′ (reverse) (Integrated DNA Technologies, Belgium). The PCR reaction was performed in 10 µL reaction volume at 95 °C for 4 min followed by 35 cycles of 95 °C for 30 s, 56 °C for 30 s and 72 °C for 15 s by a T1 Thermocycler PCR device (Biometra®, Germany). PCR of actin using RNA samples that had not been reverse transcribed was also performed to check for genomic DNA contamination (i.e. no RT control). PCR product was checked by agarose gel-electrophoresis using 2% gel (#16500-100; Thermo Fisher Scientific) and GeneRuler™ 100 bp Plus DNA ladder (#SM0328, Thermo Fisher Scientific).

### PACAP-38 peptide synthesis

The synthesis of PACAP-38 peptide was performed using a solid-phase procedure with Fmoc-chemistry. Peptide chains were elongated on a Tentagel S-Ram resin (0.23 mmol/g) and the synthesis was performed using a CEM Liberty Blue machine. The peptide was detached from the resin by using a mixture containing 90% TFA, 4% water, 2% dithiothreitol, 2% triisopropylsilane, and 2% p-cresol. The resulted crude peptide was purified by reverse-phase HPLC using a Phenomenex Luna C18 (250 × 21.2 mm, 100 Å, 10 μm) column. The appropriate fractions were pooled and lyophilized (purity > 98%) and analyzed using liquid chromatography-electrospray ionization-mass spectrometry.

### Immunohistochemistry

The heart was dissected from individual snails (*n* = 5) and pinned out on a Sylgard-coated dish containing 4% paraformaldehyde in 0.1 M phosphate buffer (pH = 7.4) overnight at 4 °C. After washing with phosphate-buffered saline (PBS; 137 mM NaCl, 10 mM Na_2_HPO_4_, 1.8 mM KH_2_PO_4_, pH = 7.4), fixed tissues were cryoprotected in 20% glucose solution for 4 h at room temperature and embedded into Cryomatrix (#6,769,006, Thermo Fisher Scientific). Series of 12–14 μm-thick cryostat sections were mounted onto slides (#J3800AMNZ, Thermo Fisher Scientific). Next, the samples were incubated with PBS containing 0.25% TritonX-100 and 0.25% bovine serum albumin (BSA) (PBS-TX-BSA) for 1 h at 4 °C.

The sections were incubated with a sheep polyclonal anti-human PACAP-38 antibody (#ab35342, Abcam) diluted 1:1000 in PBS-TX-BSA or a rabbit anti-PAC_1_ receptor antibody (from Showa University, Tokyo, Japan; antigen: a synthetic peptide corresponding to the C-terminal intracellular domain conjugated to hemocyanin from giant keyhole limpet [KLH]) diluted 1:2000 in PBS-TX-BSA for 24 h at 4 °C. After the incubation, the samples were washed with PBS for 2 × 15 min. The PACAP-38-sections were further incubated with a rabbit anti-sheep IgG secondary antibody conjugated with horseradish peroxidase (HRP, #ab97130, Abcam) diluted 1:1000 in PBS-TX-BSA overnight at room temperature. The immunoreaction was visualized by adding 0.05% 3,3-diaminobenzidine (DAB) as chromogen and 0.01% H_2_O_2_ as substrate. The development was monitored under a stereomicroscope and stopped by changing the developing solution for 0.1 M Tris–HCl buffer. The PAC_1_ receptor-sections were further incubated with a donkey anti-rabbit IgG secondary antibody conjugated with NorthernLights™ NL557 (#NL004, R&D System) diluted 1:200 in PBS-TX-BSA overnight at room temperature. Finally, the sections were washed in PBS and mounted into mounting medium (DAKO, Glostrup, Denmark). The stained tissues were analyzed using a light and fluorescent microscope. Because the primary antibodies were generated against an antigen conjugated with KLH, to abolish cross-reaction to hemocyanin in tissues of *L. stagnalis*, the antibodies were preadsorbed overnight with KLH (#H7017, Merck). The specificity of the antibodies was tested applying method control and preadsorption test. In the case of method control, BSA was used instead of primary antibodies. In preadsorption tests, the diluted antibodies were mixed with their blocking peptides (synthetic human PACAP-38, 200 µg/mL) or PAC_1_-R blocking peptide (50 µg/mL, #BLP-VR003, Alomone labs) and were shaken overnight. Immunostaining was not present either in case of method control experiments or preadsorption tests.

### Western blot analysis

The hemolymph (200 µL/animal; *n* = 5) was collected in a pre-cooled tube following the standard invasive protocol (Boisseaux et al. 2016; Hernadi et al. 2008) and stored on ice until use. The whole CNS (*n* = 10) and the heart (*n* = 5) were dissected from the animals, pooled, and extracted following a previously published protocol (Fodor et al. 2022; Hernadi et al. 2008). Briefly, the tissue samples were homogenized in 500 µL lysis buffer (1 M TRIS (pH = 6.8), 2% SDS, 10% glycerol, 1% mercaptoethanol, 10% protease inhibitory cocktail (#P2714-1BTL, Merck) with a Polytron PT 1200 homogenizer (Kinematica AG) and then further extracted with ultra-sonication. After centrifugation of the homogenates and the hemolymph (16,000×g for 5 min at 4 °C), the supernatants were placed in a new tube. 160 µL of the supernatants were diluted with 5x sample buffer (0.1 M TRIS (pH = 6.8), 0.5 M DTT, 2% SDS, 10% glycerol, 1 mM EDTA, 0.1% bromophenol blue) and denatured for 5 min at 95 °C.

For the WB analysis, following a previously published protocol (Fodor et al. 2022; Hernadi et al. 2008), 5 µL samples were run three times on a 10% SDS-PAGE, each with 3 µL protein marker. In the parallel SDS-PAGE, 20 µL samples were run three times for the MS analysis. The gel then was blotted for 3 h onto a nitrocellulose membrane (#GE10600002, Merck) following the standard semi-dry blotting protocol. The parallel gel was stained with Coomassie brilliant blue following the standard protocol. The membrane was incubated for 3 h with a blocking solution (10% non-fat dry milk powder and 6% BSA in Tris-buffered saline (TBS; 20 mM Tris, 150 mM NaCl, pH = 7.6), then washed thoroughly (3 × 5 min in TBS with 1% Tween-20 (TBST), then 3 × 5 min in TBS). The membrane was then incubated with the anti-PACAP-38 (#ab35342, Abcam) diluted 1:2000 in TBST-6% BSA overnight at 4 °C. After washing (3 × 5 min in TBS with 1% Tween-20, then 3 × 5 min in TBS), the membranes were incubated with a rabbit anti-sheep IgG secondary antibody conjugated with HRP (#ab97130, Abcam) diluted 1:5000 in TBS-Tween-20-BSA for 3 h at room temperature. After washing (3 × 5 min in TBST, then 3 × 5 min in TBS), the bands were visualized using enhanced chemiluminescence (Millipore). Synthetic PACAP-38 was used to the positive control experiments. In the negative control experiments, the samples were only probed with the secondary antibody and yielded no bands. In some cases, the anti-PACAP-38 antibody was preadsorbed with 10 µM of synthetic human PACAP-38 peptide overnight at 4 °C. This resulted in no bands (not shown).

### Immunoprecipitation

The whole CNS (*n* = 15) was dissected from the animals, pooled, and homogenized in 1 mL lysis buffer (8 M urea, 20 mM TRIS-HCl pH = 8.0, 1 mM EDTA, 150 mM NaCl, 1% Triton X-100, 1 tablet of protease inhibitor cocktail (#11,836,170,001, Merck)) with a Dounce homogenizer and then further extracted with ultra-sonication. To remove urea and gain clean polypeptides and proteins, a chloroform-methanol total protein precipitation was performed. After centrifugation (16,000×g for 10 min at 4 °C), the supernatant was removed. The pellet was washed with methanol to remove the chloroform below the pellet. After centrifugation (16,000×g for 10 min at 4 °C), the pellet was resuspended in 1 mL buffer 1 (20 mM TRIS, 150 mM NaCl, 0.5% Triton X-100, pH = 7.5).

The magnetic immunoprecipitation was performed with a 12-Tube Magnet rack (#36,912, Qiagen). 200 µL suspensions of Protein A/G-conjugated magnetic beads (#88,802, Thermo Fisher Scientific) were placed in new tubes and the beads were fixed in the wall towards the magnet for 5 min. The containing buffer was removed and the fixed beads were washed with PBS three times. The anti-human PACAP-38 primary antibody diluted 1:500 in TBST-6% BSA (1 mL) was incubated with the magnetic bead – Protein A/G complexes for 1 h at room temperature with shaking. After the removal of the primary antibody solution, the whole resuspended sample (1 mL) was incubated with the magnetic bead – Protein A/G – primary antibody complexes for 2 h at room temperature with shaking. To check the reliability of the method, the procedure was also performed with 1 mL synthetic human PACAP-38 peptide solution (100 µg/mL) (positive control). The immunoprecipitated samples were washed one time with Buffer 1, two times with buffer 2 (20 mM TRIS, 150 mM NaCl, pH = 7.5), and two times with Buffer 3 (5 mM TRIS, pH = 7.5). Finally, the samples were eluted with 200 µL 1 M NH_4_OH and lyophilized.

### HPLC-MS measurement

The lyophilized samples were re-suspended in a 6 M urea solution. Before the overnight enzymatic digestion with Trypsin/Lys-C Mix (#V5072, Promega, the USA), the samples were reduced and alkylated following the manufacturer’s technical bulletin. After the digestion, the peptides were cleaned on an Oasis HLB 96-well plate (HLB 30 mg, Waters, the USA), and the eluted samples were concentrated with an Eppendorf Concentrator plus system (Eppendorf, Hamburg, Germany). The concentrated peptides were resolved in 100 µL water containing 0.1% formic acid.

Nanoseparation-based proteomics analysis was performed with a Bruker Maxis 4G UHR-QTOF instrument (Bruker Daltonics, Bremen, Germany) coupled with Bruker EASY-nLC equipment. Three µL aliquots of the samples were injected and separated on a homemade C18 analytical column (3 μm, 75 μm x 150 mm) using a multistep gradient elution at a flow rate of 250 nL min^− 1^. We used the following eluent composition for the separation: A (aqueous formic acid solution: 0.1%) and B (acetonitrile/formic acid: v/v 99.9/0.1%). The scanning range was 300–2.200 m/z. The flow of nebulizer gas was 4 L min^− 1^ on 0.6 bar and the temperature was set at 200 °C. The capillary voltage was 4.5 kV, and the top 30 peptides were fragmented with a CID fragmentation cell.

For polypeptide/protein identification, the raw data were first processed with Data Analysis 4.4 software and then the fragment spectra were analyzed with the Mascot server V2.4.1 database search engine on the Swiss-Prot database or the given sequences we specified. Searching parameters were set to allow one missed cleavage site, accepting 50 ppm mass errors at the MS^1^ and 0.3 Da at the MS^2^ mode. For the polypeptide/protein identification, we used variable modification, including methionine oxidation and carbamidomethylation on cysteine as a fixed modification.

### cAMP measurements in the heart

The heart (Supplementary Fig. 1) was dissected from individual snails (*n* = 10 animals/replicates) and pinned out on a Sylgard-coated dish containing *Lymnaea* physiological saline (50 mM NaCl, 2 mM KCl, 4 mM CaCl_2_, 4 mM MgCl_2_, 10 mM TRIS; pH = 7.5). The hearts were divided into control and synthetic PACAP-38-treated experimental groups (resulting in *n* = 5 animals/group/replicates). Based on a previously published protocol (Pirger et al. 2008, 2010b), specimens of the synthetic PACAP-38-treated group were first pre-incubated in *Lymnaea* physiological saline containing 0.5 mM 3-isobutyl-1-methyl-xanthine (#I5879, Merck) and 0.5 mM adenosine-triphosphate (#A9187, Merck) for 5 min, then incubated in *Lymnaea* Ringer containing 10 µM synthetic PACAP-38 for 10 min. The concentration of the PACAP-38 solution was chosen based on our preliminary results and our previous studies (Pirger et al. 2008, 2010a, b): the effects of vertebrate PACAP peptides have been investigated on snails at a concentration range of 10^− 5^ M − 10^− 7^ M with all concentrations were able to induce many physiological changes. In the present study, we chose the 10 µM (10^− 5^ M) concentration for a robust effect.

After the incubation, the hearts were pooled in both experimental groups, homogenized in cold acidic ethanol with a Polytron PT 1200 homogenizer (Kinematica AG), and centrifuged at 10,000×g for 10 min at 4 °C. The supernatants were collected, lyophilized (Vacuum Freeze Dryer, #BK-FD10S, Biobase), and dissolved in Tris–EDTA buffer. After centrifugation at 16,000×g for 10 min at 4 °C, the cAMP content was determined by the protein binding method using the Amersham cyclic AMP assay system (GE Healthcare UK Limited), according to the manufacturer instructions. Experiments were performed in 3 independent series.

### Pharmacological experiments

For pharmacological experiments, we used the heart of *L. stagnalis* which is an excellent organ for studying chemical modulation (reviewed by Benjamin (Benjamin 2008). Its physiology is well-known: while heartbeat is generated by a muscle pacemaker located in the heart, its regulation is basically implemented by many neurotransmitters and neuropeptides which are released by several types of motoneurons into the heart (Benjamin and Kemenes 2013, 2020; Buckett et al. 1990a, b, c; Santama and Benjamin 2000; Willoughby et al. 1999a, b; Worster et al. 1998). All pharmacological experiments were carried out on isolated heart preparations using a previously developed refraction-based optical recording system (Pirger et al. 2023).

A three-way tap ensured rapid switching with minimum mixing of perfusates. The pressure heads containing *Lymnaea* physiological saline, 7 µM serotonin (5-HT) (#H9523-25MG, Merck), 2.5 nM acetylcholine (ACh) (#A6500-100G, Merck), and 10 µM synthetic PACAP-38 solutions were kept l-2 cm above the level of the heart. This was required for maintaining a steady beat. The test solutions were made just before the test. The concentration of 5-HT and ACh solutions was chosen based on the pharmacology of the heart of *L. stagnalis* described by previous studies (Benjamin 2008; Buckett et al. 1990a). Similarly to the cAMP measurement, the concentration of the PACAP-38 solution was chosen for a robust effect. Perfusions of test substance were of 1-min durations, *Lymnaea* physiological saline being perfused between each application until the heart had returned to a steady beat rate. The period of this normal saline perfusion varied according to the effects of the previous substance application but was never < 4 min.

Experiments were carried out to explore whether synthetic PACAP-38 had any direct or modulatory effect on heart physiology. To investigate the possible direct effect on the average amplitude of heart muscle contraction, 10 µM synthetic PACAP-38 solution was directly applied to the heart preparations (*n* = 16). To investigate the possible modulatory effects on the heart muscle responses (tonus, amplitude of contraction, relaxation), based on our previous experiments (Krajcs et al. 2015), the heart preparations (*n* = 6) were pre-treated with 10 µM synthetic PACAP-38 solution for 15 min before the direct application of 5-HT and ACh. The 7 µM 5-HT solution is known to increase both beat rate and amplitude, while 2.5 nM ACh solution is known to decrease both beat rate and amplitude (Buckett et al. 1990a).

### Statistical analysis

Statistical analysis was carried out using OriginPro8 2018 software (OriginLab Corp., Northampton, Massachusetts, USA). The normality of the dataset was investigated using the Shapiro-Wilk test, homogeneity of variances between groups was investigated using the F-test. In the case of cAMP level measurements, differences between groups were analyzed by a two-sample t-test. In the case of average amplitude of contraction measurements, differences were analyzed by a paired t-test. In the case of 5-HT and ACh response experiments, comparisons between more than two independent groups were carried out using one-way ANOVA followed by multiple post hoc Tukey’s tests. Differences were considered statistically significant at least *P* < 0.05 (*^,#^). As a bias-reducing effort, the analysis of the data generated during the biochemical and pharmacological experiments was blinded.

## Results

### No homologous sequences to the vertebrate PACAP and its receptors

The sequence searches in the *L. stagnalis* neuronal transcriptome and genome data failed to identify putative transcripts or genes to the elements (precursor, active peptides, receptors) of the vertebrate PACAP system or the cephalochordate PACAP/GCG system (Table 1). Moreover, any homologous sequences to other peptides of the Secretin superfamily were not found either (Supplementary Table 5).

**Table 1:** *L. stagnalis* homologs to sequences encoding vertebrate PACAP peptides and their receptors

	Peptide		Receptor		
	**prepro-PACAP**	**PACAP-27/38**	**PAC**_**1**_	**VPAC**_**1**_	**VPAC**_**2**_
Homologous gene/transcript in *L. stagnalis*	not found	not found	not found	not found	not found

### An anti-human PAC_1_ receptor antibody yielded a positive signal in the heart

**Fig. 1:**
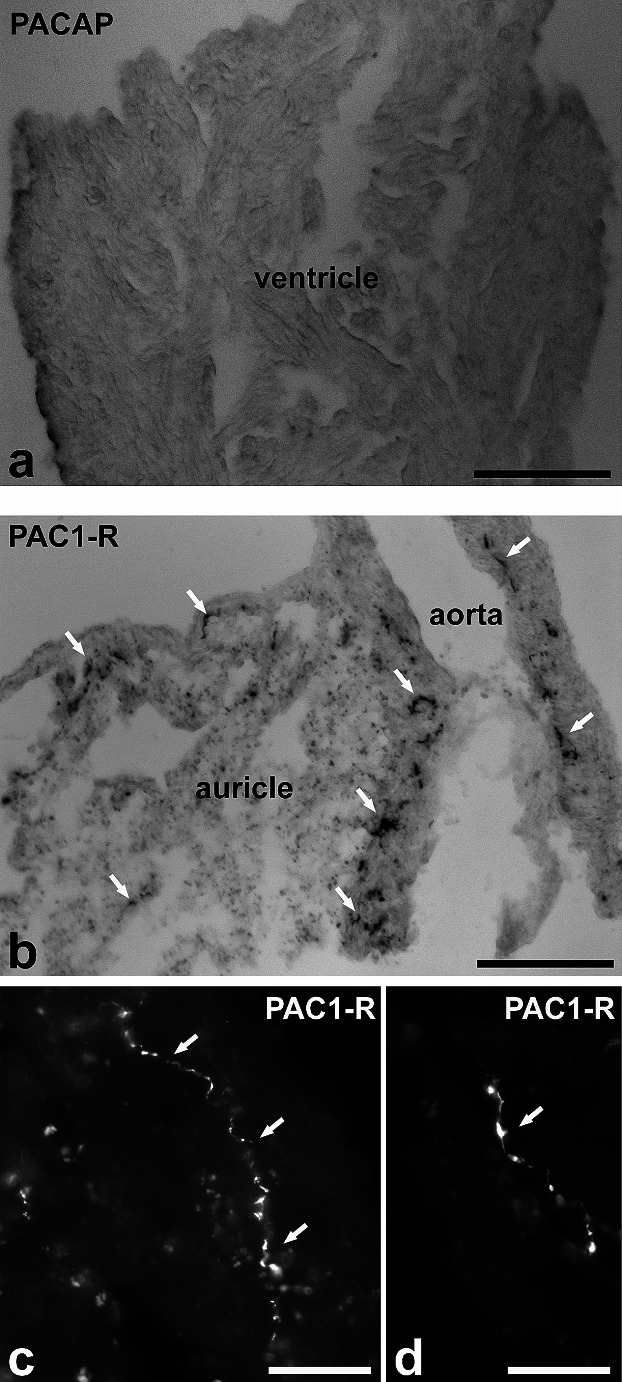
Representative PACAP-38 and PAC_1_ receptor immunolabeling in the heart. **a** The anti-human PACAP-38 antibody gave no immunoreactivity. The anti-human PAC_1_ receptor antibody yielded immunopositive fine varicose fibers (arrows) in both **b**,**c** the auricle and **d** the ventricle. Scale bars = 0.4 mm in **a**, 0.3 mm in **b**, 100 μm in **c**,**d**

Our IHC revealed no vertebrate PACAP-38-immunopositive neuronal elements in the heart (Fig. 1a). In contrast, despite the lack of the homolog sequence to the vertebrate PAC_1_ receptor in *L stagnalis*, the antibody corresponding to the C-terminal region of human PAC_1_ receptor identified immunopositive varicose fibers in both the auricle (Fig. 1b, c) and the ventricle (Fig. 1d). The heart muscle fibers themselves were not immunopositive.

### Investigation of polypeptide(s) labeled by the human anti-PACAP-38 antibody

**Fig. 2:**
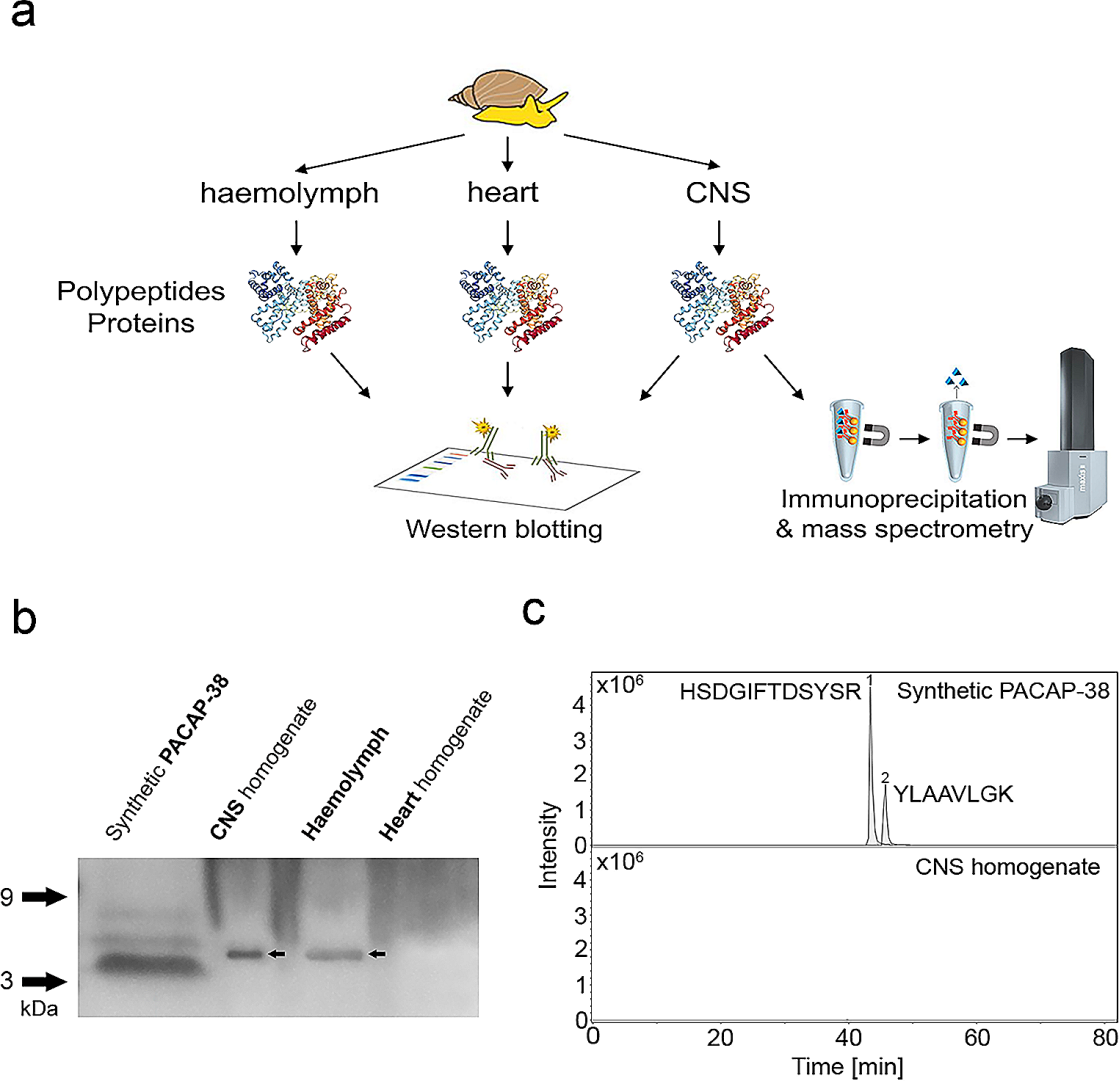
Characterization of the polypeptide(s) that showed cross-reactivity with the anti-human PACAP-38 antibody. **a** Schematic representation of the methodical approaches to characterize the marked polypeptide(s). **b** Western blot analysis of the homogenate of the CNS, hemolymph, and the homogenate of the heart. The antibody yielded one discrete band (∼ 5 kDa) in the columns containing the extract of CNS and hemolymph. Using synthetic PACAP-38, the typical ∼ 4.5 kDa band was detected. **c** Extracted ion chromatogram of the positive control containing synthetic PACAP-38 (top panel) and the homogenate of the CNS (bottom panel). The main characteristic peptide fragments (1: HSDGIFTDSYSR; 2: YLAAVLGK) of PACAP-38 were identified in the positive control, but they were not detected in the homogenate of the CNS

Despite the lack of the homologous sequence to the vertebrate PACAP-38 in *L. stagnalis*, our previous IHC investigations with an anti-human PACAP-38 antibody yielded a positive signal in the CNS (Pirger et al. 2010b). To characterize the polypeptide(s) labeled by the vertebrate antibody (cross-reactivity), we first performed PAGE and WB analysis on the homogenate of the CNS, hemolymph, and the homogenate of the heart (Fig. 2a, b).

**Fig. 3:**
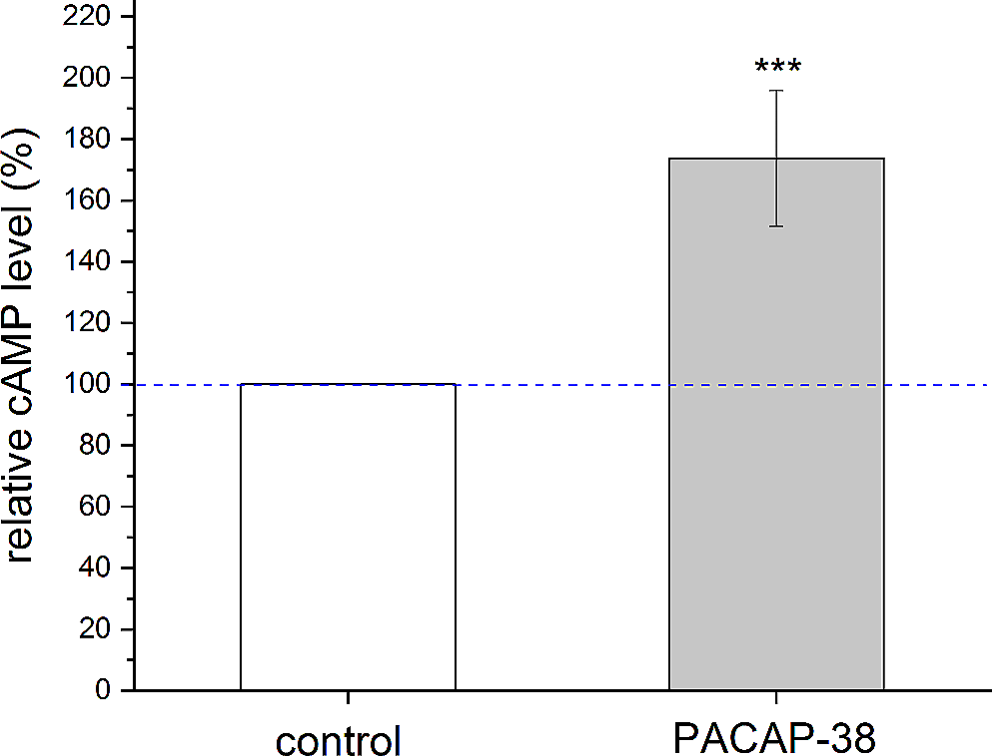
Effect of 10-min incubation with 10 µM synthetic PACAP-38 peptide on cAMP synthesis in homogenates of *L. stagnalis* heart. Data are presented as mean % ± SEM, normalized to the mean of the control group (100%, blue dashed line) (*n* = 5 animals/group/replicates). Compared to the control group, the 10 µM PACAP-38 treatment significantly increased the cAMP levels (*P < 0.05)

**Fig. 4:**
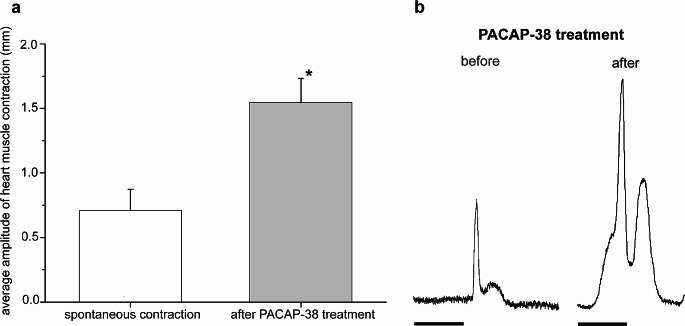
Direct effect of 10 µM PACAP-38 peptide on the heart muscle. **a** Average amplitude of heart muscle contraction calculated by measuring the amplitude of seven random peaks for 1 min before and after the PACAP-38 treatment. Each bar represents mean ± SEM (*n* = 16). White and grey columns represent the score before and after treatment, respectively. Compared to the control group, the 10 µM PACAP-38 treatment significantly increased the average amplitude of heart muscle contraction (*P < 0.05). **b** Representative random peaks measured before and after the PACAP-38 treatment. Scale = 3 s

**Fig. 5:**
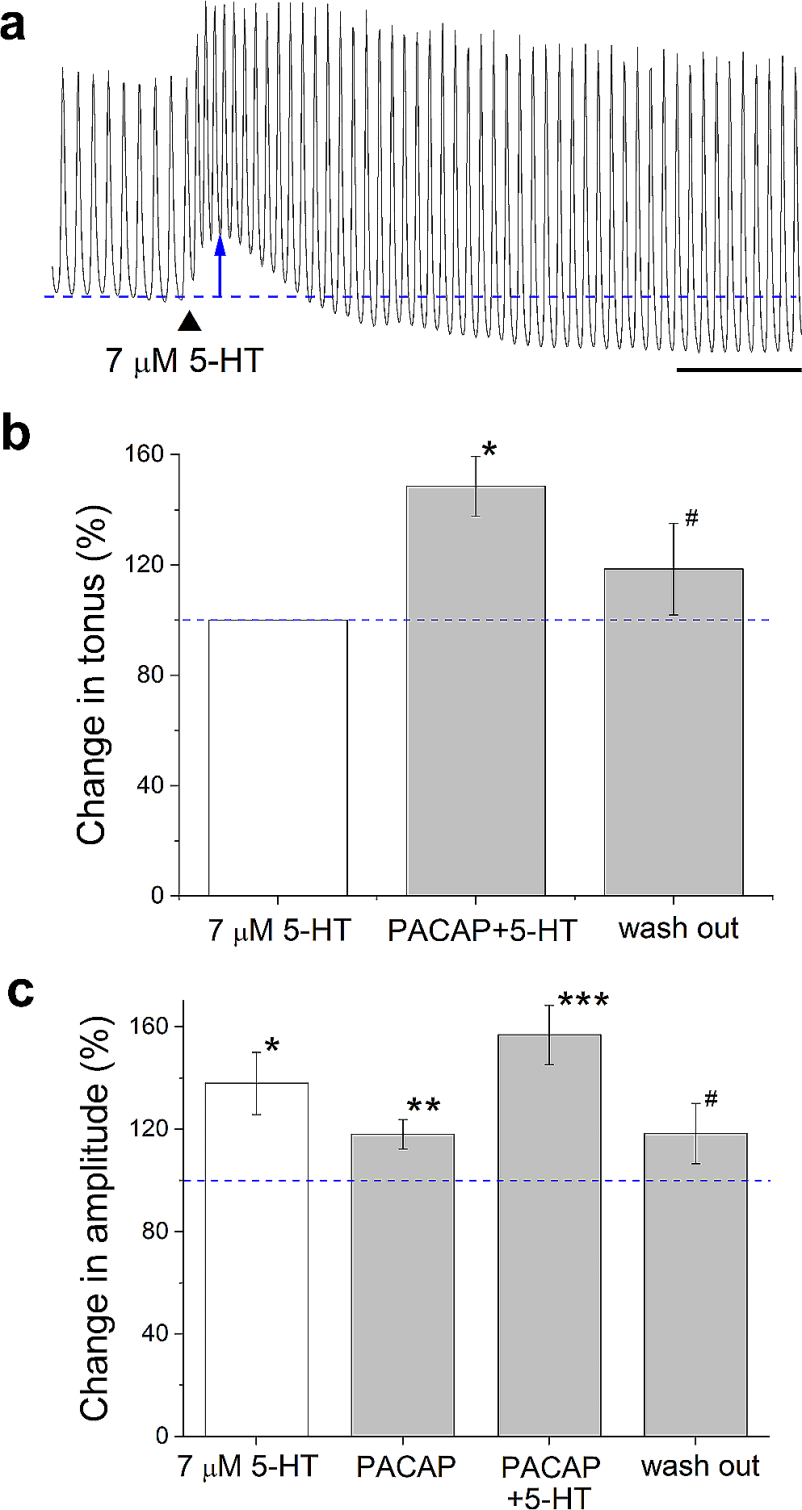
PACAP-38 enhanced the effect of 5-HT on the heart muscle. **a** Effect of 7 µM 5-HT on the atrioventricular contraction: heart rate, amplitude, and tonus increased (scale = 60 s). **b** Pretreatment with 10 µM PACAP significantly enhanced the effect of 5-HT on the basal tonus (*P < 0.05). After 4 min wash out, the tonus returned close to the control (^#^*P* < 0.05, compared to PACAP + 5-HT). Data are presented as mean % ± SEM, normalized to the mean tonus caused by 5-HT (100%, blue dashed line) (*n* = 6 animals/group/replicates). **c** 5-HT (*P < 0.05) or pretreatment with 10 µM PACAP (*P < 0.01) or 5-HT + PACAP (*P < 0.001) significantly increased the basal amplitude. After 4 min wash out, the amplitude returned close to the control (^#^*P* < 0.05, compared to PACAP + 5-HT). Data are presented as mean % ± SEM, normalized to the normal (basal) mean amplitude (100%, blue dashed line) (*n* = 6 animals/group/replicates)

**Fig. 6:**
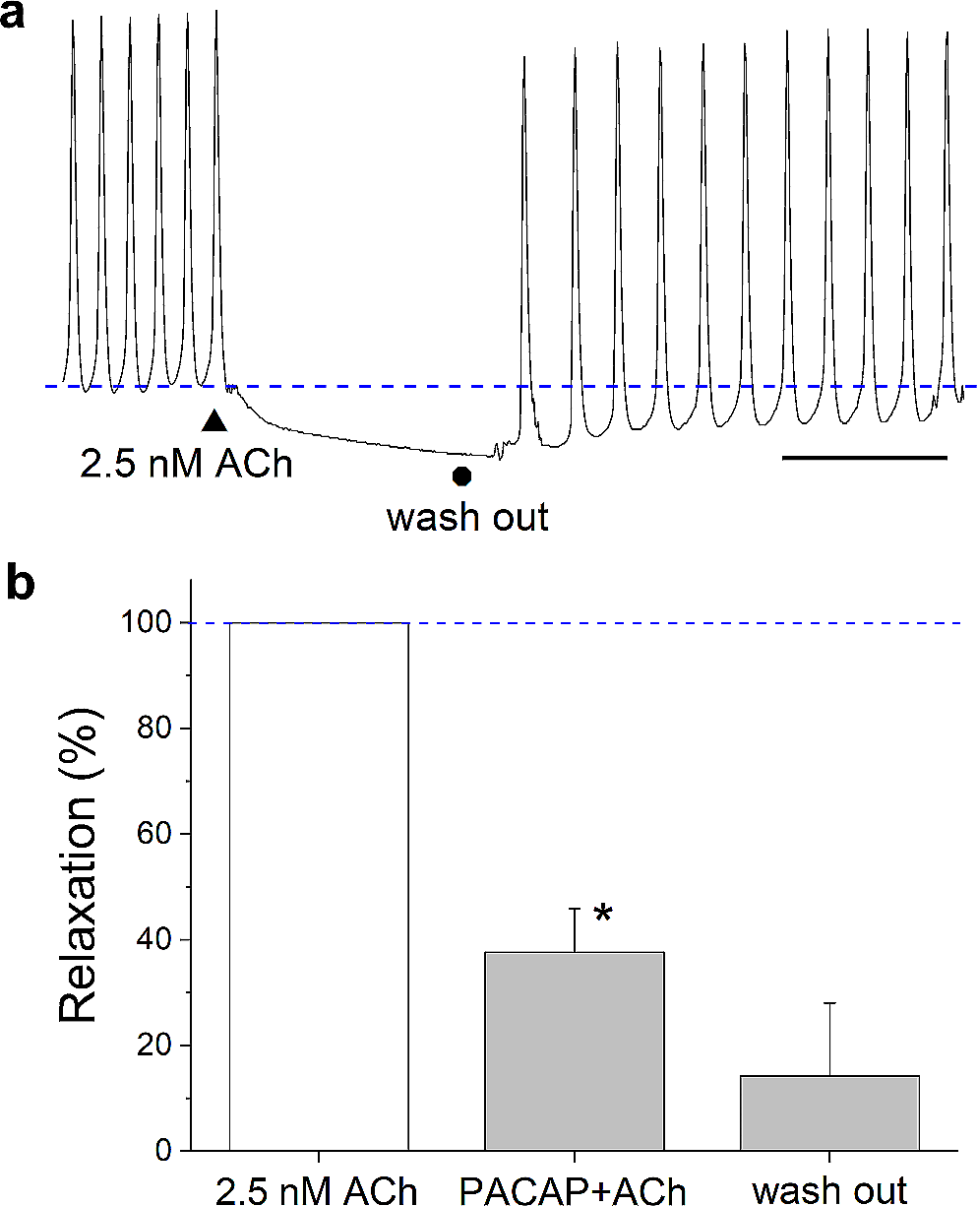
PACAP-38 offset the effect of ACh on the heart muscle. **a** Effect of 2.5 nM ACh on the atrioventricular contraction, both heart rate and amplitude were reduced (scale = 60 s). **b** Pretreatment with 10 µM PACAP significantly compensated the inhibitory effect of ACh (*P < 0.05). After 4 min wash out, the tonus returned close to the basal value. Data are presented as mean % ± SEM, normalized to the mean relaxation caused by ACh (100%, blue dashed line) (*n* = 6 animals/group/replicates)

In the positive control experiment, the antibody targeting the synthetic human PACAP-38 peptide yielded three distinct bands with one at ∼ 4.5 kDa corresponding to the molecular weight (4534.26) of human PACAP-38. In columns containing CNS homogenate and hemolymph, a discrete ∼ 5 kDa band with positive immunoreaction was obtained. In contrast, corresponding to the IHC data presented above, immunoreaction was not observed in the column containing the heart homogenate.

To determine the non-specifically labeled polypeptide(s), immunoprecipitation and MS analysis were also performed on the homogenate of the CNS (Fig. 2a, c). In the positive control experiment, the main characteristic peptide fragments of synthetic PACAP-38 were clearly identified: (1) HSDGIFTDSYSR (1383.62 m/z, [M]^2+^) and (2) YLAAVLGK (834.508 m/z, [M + H]^+^) (Fig. 2c top panel; Supplementary Fig. 2), confirming the reliability of the immunoprecipitation method. In contrast, these characteristic peptide fragments were not detected in the homogenate of the CNS (Fig. 2c bottom panel), further confirming that PACAP-38 is not present in the CNS of *L. stagnalis*. Although several peptide fragments were present in the tissue sample (Supplementary Fig. 3), which did not correspond to the peptide fragments of synthetic PACAP-38, the polypeptide(s) that showed cross-reactivity with the anti-human PACAP-38 antibody remain to be determined.

### PACAP-38 increased the cAMP level in the heart

Figure 3 presents the effect of a 10-min incubation with 10 µM synthetic PACAP-38 on the cAMP synthesis in the heart showing that an approximately 74% increase (173.7 ± 22.1 of control level) in cAMP levels was obtained (*P* < 0.05).

### Direct and modulatory effects of PACAP-38 on heart muscle

The direct application of 10 µM PACAP-38 peptide significantly increased the mean amplitude of heart muscle contraction (*P* < 0.05) (Fig. 4). To reveal whether synthetic PACAP-38 had also a modulatory effect, 5-HT (7 µM) was first directly applied to the heart muscle resulting in the increase of both heart rate and amplitude (Fig. 5a). Pretreating the muscle with 10 µM synthetic PACAP-38 for 15 min induced a significant enhancement of the 5-HT evoked effect on both of the tonus (*P* < 0.05) (Fig. 5b) and the amplitude (*P* < 0.001) (Fig. 5c). Next, ACh (2.5 nM) was directly applied to the heart muscle resulting in the reduction of both heart rate and amplitude (Fig. 6a). Pretreating the muscle with 10 µM synthetic PACAP-38 for 15 min significantly compensated the inhibitory effect (relaxation) of ACh (*P* < 0.05) (Fig. 6b).

### Presence and expression of members of the cluster B receptor subfamily

**Fig. 7:**
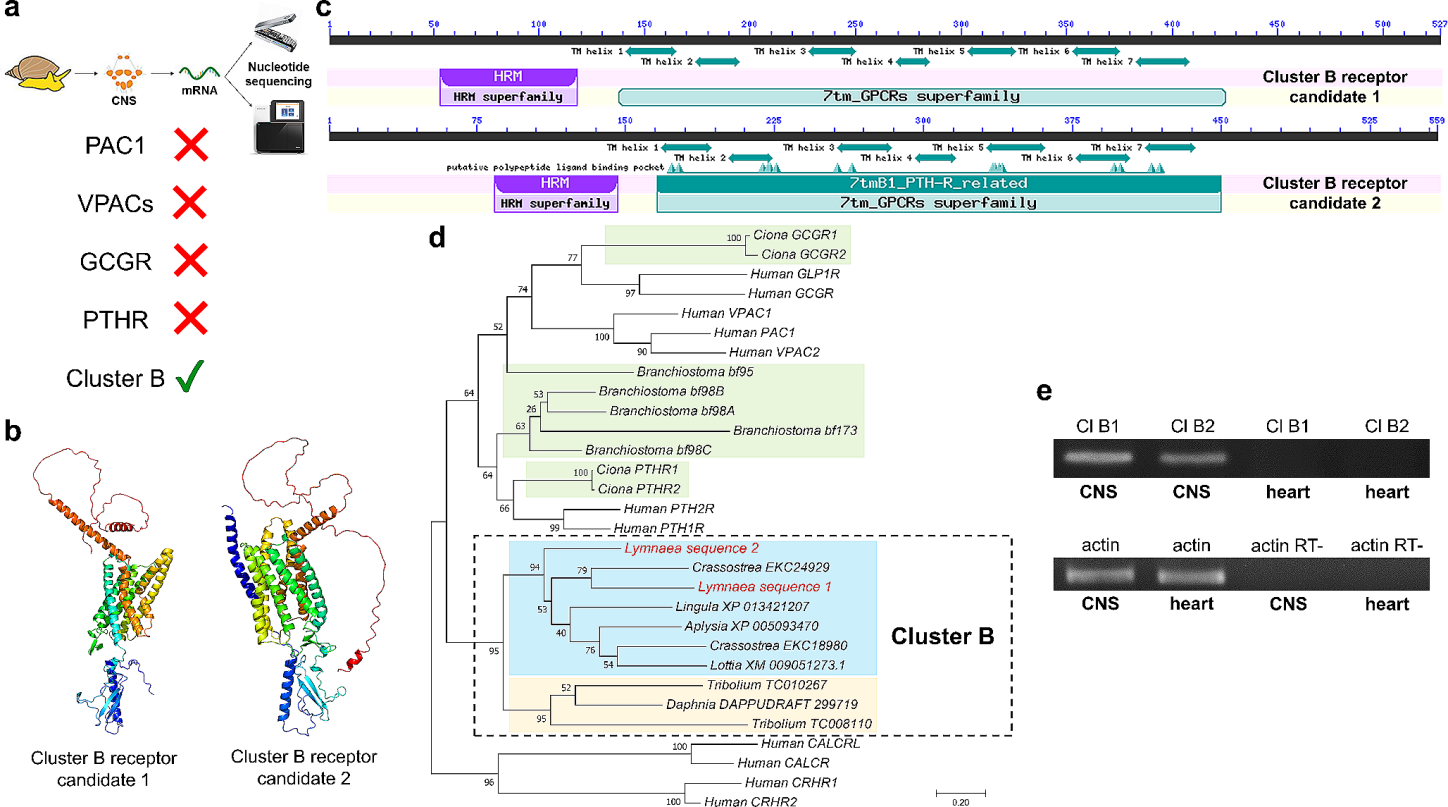
Identification, *in silico* analysis, and expression of *L. stagnalis* Cluster B receptors. **a** Presence of homologous sequences in the neuronal transcriptome of *L. stagnalis* to the members of Cluster B subfamily of the B1 GPCR family. **b** Predicted 3D structure of *L. stagnalis* Cluster B receptors. **c** Conserved domain analysis of *L. stagnalis* Cluster B receptors. **d** Phylogenetic tree of the protostome Cluster B receptors and the deuterostome PAC1, VPAC_1_, VPAC_2_, GCGR, and PTHR members showing that the obtained *L. stagnalis* sequences clearly and robustly group with other known molluscan Cluster B receptors. Cephalo/urochordate, molluscan, and arthropod sequences are highlighted with green, blue, and orange color, respectively. The *L. stagnalis* sequences are highlighted with red. **e** PCR of cDNA synthesized from the CNS and heart. Top panel: PCR of Cluster B receptor 1 (Cl B1, 461 bp) and Cluster B receptor 2 (Cl B2, 449 bp) receptors. Bottom panel: PCR of actin (140 bp) to check the quality of RNA samples and PCR of actin using RNA samples that have not been reverse transcribed to check for genomic DNA contamination (actin RT-)

Although the *in silico* searches yielded no direct sequence homologs of vertebrate PAC_1_, VPAC_1_, and VPAC_2_ receptors or the cephalochordate PACAP/GCG receptor (generally referred to as bf95), they retrieved two homologous sequences to the members of Cluster B subfamily of the B1 GPCR family (Fig. 7a; Supplementary Fig. 4). The identified sequences showed relatively low conservation with other protostome and deuterostome B1 GPCRs (Supplementary Table 6). The predicted 3D structure (Fig. 7b) and the conserved domain analysis (Fig. 7c; Supplementary Fig. 5) of the proteins suggested that these newly identified *L. stagnalis* Cluster B receptor candidates are likely functional. Moreover, the phylogenetic analysis showed that the obtained *L. stagnalis* sequences clearly and robustly group with other known molluscan Cluster B receptors (Fig. 7d), thus supporting their homology. Reverse transcription (RT) polymerase-chain reaction (PCR) revealed that both Cluster B receptors were expressed in the CNS but not in the heart (Fig. 7e).

## Discussion

Although several papers have been published in the last decades claiming the presence of a homolog of the vertebrate PACAP systems in protozoan, non-bilaterian, and protostome species, the robustness of the IHC and partial sequence data in most of these papers recently has been questioned (Cardoso et al. 2020). Despite the active peptide sequence(s) being theoretically highly conserved among taxa (Supplementary Fig. 6), we did not find any homologous sequences to vertebrate genes coding for PACAP or its receptors during the screening of our *L. stagnalis* transcriptome and genome data or the publicly available *L. stagnalis* sequence data. This supports the idea that these classical vertebrate sequences are absent in mollusks and that elements of the PACAP system emerged after the protostome-deuterostome divergence (Cardoso et al. 2010, 2020; On and Chow 2016; On et al. 2022). As a supplementary investigation, we also searched for homologous sequences to other members of the Secretin neuropeptide superfamily, but this analysis also yielded no hits in our data or in the public data. This confirms the previous notion that the entire Secretin neuropeptide superfamily appeared in vertebrate genomes (Cardoso et al. 2020). In our opinion, supported by a recent review of Cardoso and his co-workers (Cardoso et al. 2020), both the previously reported non-bilaterian and protostome partial cDNA sequences encoding a peptide highly similar/identical to vertebrate PACAP and the previously reported MS results on the presence of molluscan PACAP peptides may have been artifacts (e.g., standard contamination in the case of MS analysis).

In vertebrates, PACAP is most abundant in the hypothalamus (Arimura et al. 1991; Ghatei et al. 1993; Hirabayashi et al. 2018; Valiante et al. 2006), but also occurs in numerous parts of the central nervous system and peripheral organs (Toth et al. 2020). PACAP receptors also have a wide distribution in different organs including the brain, eyes, heart, lung, liver, pancreas, testis, and the breast (Lu et al. 2022; Nakamachi et al. 2016; Szanto et al. 2012; Toth et al. 2020; Vaudry et al. 2009). As mentioned in the Introduction section, another key line of evidence that, on the surface, appears to support that a homolog of the vertebrate PACAP system is present protostomes is that antibodies raised against vertebrate PACAP peptides and their receptors bind to cells in their tissues (Boros et al. 2008, 2010; Hernadi et al. 2008; Krajcs et al. 2015; Molnar et al. 2006, 2008; Pirger et al. 2008, 2010b; Reglodi et al. 2000; Somogyi et al. 2009; Varhalmi et al. 2008; Zhong and Pena 1995). Such positive staining has been claimed as evidence for the presence of an invertebrate PACAP-like peptide despite these genes not being found in the genome of any species outside chordates (Cardoso et al. 2020). Although these genes are not present in *L. stagnalis* either, like the other studies, we also found positive staining with an anti-human PACAP-38 antibody in the CNS in one of our previous studies (Pirger et al. 2010b) and with an anti-human PAC_1_ antibody in the heart in the present study. The positive signal in the heart of *L. stagnalis* would not be fundamentally new information because the same observation was previously published in the case of the Burgundy snail (*Helix pomatia*) (Hernadi et al. 2008). However, this finding and the previous IHC results (e.g., positive signal in the CNS for PACAP) mean something completely different in light of the absence of the homologous genes to the elements of the vertebrate PACAP system: one should again question the meaningfulness of positive immunostaining with vertebrate antibodies for identifying or localizing specific polypeptide(s)/proteins in molluscan tissues (and in invertebrates in general) (Cardoso et al. 2020; Fodor et al. 2022).

To characterize the polypeptide(s) that showed cross-reactivity with the anti-human PACAP-38 antibody, we first performed WB analysis on the homogenate of the CNS, hemolymph, and the homogenate of the heart. Previously, a WB analysis of the CNS homogenates of *H. pomatia* with anti-human PACAP-27 and anti-human PACAP-38 antibodies revealed a positive signal: one discrete band at ∼ 4.5 kDa corresponding to the band of human PACAP-38 (Hernadi et al. 2008). At that time, this corresponding labeling confirmed the assumption about the presence of PACAP in mollusks (due to the lack of genome/transcriptome sequence information). Similarly, our present analysis with an anti-human PACAP-38 antibody yielded one discrete band at ∼ 5 kDa. To determine the non-specifically labeled polypeptide(s), we also made an immunoprecipitation experiment. Although we could clearly verify the lack of peptide fragment sequences that were homologous to those found in vertebrate PACAP-38, we could not identify the polypeptide(s) marked by the antibody. Several peptide fragments were present in the sample, which can be attributed to the disadvantage of the method that it tends to extract other proteins in addition to the target one. Using the Swiss-Prot database, we could identify more proteins but not one whose size corresponded to the range of the labelled polypeptide observed in the WB. We did not exclude the possibility that we could not identify the polypeptide with the Swiss-Prot database due to the relatively low number of molluscan sequences included. Hence, we manually compared the peptide sequences with known *L. stagnalis* polypeptide(s) with relevant mass (Supplementary Table 7), but not a single one of them matched. We also consider it possible that the polypeptide cannot even be identified with the currently known databases, it is not certain that a polypeptide with such a low size has a cleavage site and that a characteristic fragment will be produced. Nevertheless, our results clearly confirm that the homolog of the highly conserved vertebrate PACAPs, which was previously assumed to be present in non-chordates animals as well, cannot be found in *L. stagnalis*.

As presented in the Introduction, receptor activation by PACAP induces cAMP production in vertebrates. In our biochemical measurements, incubation with 10 µM synthetic PACAP-38 significantly increased the cAMP synthesis in the homogenate of heart. This corresponds to the findings of previous studies which also demonstrated an increase in cAMP production by 10 µM PACAP-38 in the homogenate of the salivary gland of *H. pomatia* (Pirger et al. 2008) and in the homogenate of the CNS of *L. stagnalis* (Pirger et al. 2010b). Similarly, vertebrate PACAP-27/38 peptides have been shown to increase cAMP levels in the vertebrate heart (Cui et al. 2000; Sano et al. 2002; Suzuki et al. 1993). It should be highlighted that the PAC_1_ receptor antagonist PACAP-6-38 significantly reduced the cAMP-increasing effect in *L. stagnalis* (Pirger et al. 2010b).

Previous studies demonstrated that vertebrate PACAP peptides have a biphasic (both positive and negative) chronotropic and a positive ionotropic effect on the vertebrate heart (Chang et al. 2005; Hirose et al. 1997; Hoover et al. 2009, 2013; Ross-Ascuitto et al. 1993; Suzuki et al. 1993). In the case of *L. stagnalis*, we also observed a positive ionotropic effect when directly applying 10 µM synthetic PACAP-38. Pretreating the heart with 10 µM synthetic PACAP-38 resulted in a modulatory effect: the increase in both tonus and amplitude induced by 5-HT was significantly enhanced while the inhibition of ACh was significantly compensated. To the best of our knowledge, this is the first study to investigate the direct or modulatory effects of vertebrate PACAP-38 on the heart in invertebrates.

Given that the genes encoding the elements of the PACAP system are chordate-specific, one can agree that the role of vertebrate PACAP peptides is enigmatic in protostome physiology. Focusing on *L. stagnalis*, in carefully designed and executed experiments (also using randomization and blinding), systemic application of the PACAP receptor antagonist PACAP6-38 was shown to inhibit memory formation after both single-trial chemical and multi-trial tactile food-reward conditioning (Pirger et al. 2010a). Moreover, systemic application of synthetic human PACAP-38 accelerated memory formation during multiple-trial tactile food-reward conditioning while co-application of the PACAP receptor antagonist PACAP6-38 with the synthetic PACAP-38 prevented the memory boosting effect of the latter (Pirger et al. 2010a). These results appear to indicate that a protostome PACAP-like peptide, identical or highly similar to the vertebrate PACAP peptides, is necessary and instructive for the formation of associative memory in *L. stagnalis*. This idea is strengthened by other results that systemic application of synthetic human PACAP-38 reversed age-related memory impairment and also had a neuroprotective function in dopamine-based neurodegeneration developed in the *Lymnaea* parkinsonian model (Maasz et al. 2017; Pirger et al. 2014). Similarly to *L. stagnalis*, previous pharmacological studies on *H. pomatia* demonstrated physiological changes and also showed that the effects could be abolished by a receptor antagonist or other inhibitors (e.g., PKA blocker H-7) (Krajcs et al. 2015).

Despite the described physiological effects, previous results (Cardoso et al. 2020) and our molecular investigations support the idea that members of the Secretin neuropeptide superfamily including the PACAP system are not present in protostomes including *L. stagnalis*. Keeping this in mind, we believe that the physiological effects described in protostomes, no matter how similar they are to those in vertebrates, are non-specific. The apparently non-specific physiological changes are really raising the important question of what the cellular and molecular underpinnings of these changes are. A possible explanation is interactions with non-specific G-protein coupled receptors for other compounds due to low-specificity ligand-receptor interactions (Cardoso et al. 2020; Pirger et al. 2010a). This is in effect yet another example of promiscuity of another typical ‘lock and key’ mechanism, i.e., the vertebrate PACAP peptides are similar enough in structure to the natural protostome peptide ligands to be able to bind to their receptor(s). Members of Cluster B subfamily of the B1 GPCR family are considered to be the protostome orthologous sequences of vertebrate PAC_1_, VPAC_1_, VPAC_2_, glucagon receptor, and parathyroid hormone receptor, which they likely share a common evolutionary origin with (Cardoso et al. 2014, 2020, 2024). We identified Cluster B receptor homologs in *L. stagnalis* and demonstrated that they are likely functional. Our phylogenetic tree confirmed this evolutionary model proposed by Cardoso and his co-workers and supported the idea that protostome Cluster B receptors are the most similar in sequence to the vertebrate PACAP receptors. However, their expression is putatively absent in the heart which does not support a potential role in the mediation of PACAP-induced physiological effects in this organ.

## Conclusions

Overall, our findings strongly support the idea that the PACAP system is not present in mollusks and emerged after the protostome-deuterostome divergence. Our results again call into question the meaningfulness of positive immunostaining with vertebrate antibodies for identifying or localizing proteins in invertebrate tissues. Many previous studies that used IHC approaches on invertebrates based on the use of vertebrate antibodies need to be revised in light of the omics data that is becoming available for many species. We propose that Cluster B receptor subfamily members may not be involved in the mediation of the reported PACAP-induced effects in non-chordate animals. The physiological effects of vertebrate PACAP peptides in protostomes, no matter how similar they are to those in vertebrates, should be considered non-specific and may take place via one or more highly promiscuous stimulative regulative G-protein GPCRs. Of course, the next enigmatic question could be what the probability is that the specific receptor antagonists (e.g., PACAP6-27, PACAP6-38) also bind to the same non-specific receptor(s) and block the physiological effects of vertebrate PACAP peptides in mollusks.

## Electronic supplementary material

Below is the link to the electronic supplementary material.

Supplementary Material 1

## Data Availability

The datasets generated and/or analyzed during the biochemical and pharmacological experiments of the current study are available from the corresponding author upon a reasonable request. The neuronal transcriptome assembly and raw sequence data will be published in another paper and will be publicly available on NCBI accordingly.

## Declarations

### Author Statement

The authors declare that the recommendations of the ARRIVE 2.0. guideline were considered and followed during the experiments, analysis, and data reporting.
