# Supplementary material for: Same same, but different: exploring the enigmatic role of the pituitary adenylate cyclase-activating polypeptide (PACAP) in invertebrate physiology
Source: J Comp Physiol A Neuroethol Sens Neural Behav Physiol. 2024 Jun 28;210(6):909–25. doi: 10.1007/s00359-024-01706-5 (PMC11551080; doi:10.1007/s00359-024-01706-5)
Supplement: Supplementary file 1 — Supplementary Material 1 [file 359_2024_1706_MOESM1_ESM.docx]

**Supplementary information**

**Same same, but different: exploring the enigmatic role of the pituitary adenylate cyclase-activating polypeptide (PACAP) in invertebrate physiology**

^1^Zsolt Pirger, ^2^Péter Urbán, ^2^Bence Gálik, ^3^Bence Kiss, ^3^Antal Tapodi, ^3^János Schmidt, ^4^Gábor K. Tóth, ^5^Joris M. Koene, ^6^György Kemenes, ^7^Dóra Reglődi, ^1^Tibor Kiss, and ^1^István Fodor*

^1^Ecophysiological and Environmental Toxicological Research Group, HUN-REN Balaton Limnological Research Institute, 8237 Tihany, Hungary

^2^Genomics and Bioinformatics Core Facilities, Szentágothai Research Centre, University of Pécs, 7624 Pécs, Hungary

^3^Institute of Biochemistry and Medical Chemistry, Medical School, University of Pécs, 7624 Pécs, Hungary

^4^ Department of Medical Chemistry, University of Szeged, Szeged, Hungary

^5^Ecology & Evolution, Amsterdam Institute for Life and Environment, Faculty of Science, Vrije Universiteit, Amsterdam, the Netherlands

^6^Sussex Neuroscience, School of Life Sciences, University of Sussex, Brighton, BN1 9QG, UK

^7^Department of Anatomy, ELKH-PTE PACAP Research Team, Centre for Neuroscience, Medical School, University of Pecs, 7624, Pecs, Hungary

*Corresponding author; E-mail: [fodor.istvan@blki.hu](mailto:fodor.istvan@blki.hu) (I Fodor)

**Supplementary Table 1**. Members of the Secretin neuropeptide superfamily (based on ([Cardoso et al. 2020](#_ENREF_4))). The superfamily consists of two peptide families (Secretin peptide family and Glucagon peptide family).

| **Secretin peptide family** |
| --- |
| Secretin |
| Vasoactive Intestinal Peptide (VIP) |
| Peptide Histidine Isoleucine (PHI) |
| PACAP |
| PACAP-Related Peptide (PRP) |
| Growth Hormone-Releasing Hormone (GHRH) |
|  |
| **Glucagon peptide family** |
| Glucagon (GCG) |
| Glucagon-Like Peptide 1 (GLP1) |
| Glucagon-Like Peptide 2 (GLP2) |
| Glicentin |
| Glicentin-related pancreatic polypeptide (GRPP) |
| Oxyntomodulin |

**Supplementary Table 2.** Data on the presence and function of PACAP peptides and their receptors in protozoan, non-bilaterian, and protostome species. The relevant *L. stagnalis* data are highlighted with red color. Abbreviations: MS – mass spectrometry, IHC – immunohistochemistry, WB – Western blotting, CNS – central nervous system, PKA – protein kinase A, PKC – protein kinase C, AC –adenylyl cyclase

| **Taxon**  **(Species)** | **Gene for prepropeptide or receptor** | **cDNA for prepropeptide or receptor** | **MS** | **IHC** | **WB** | **Effect of PACAP-27/38 on cAMP synthesis** | **Effect of PACAP-27/38**  **on physiology** | **Effect of antagonists** | **Effect of other inhibitors** | **Reference(s)** |
| --- | --- | --- | --- | --- | --- | --- | --- | --- | --- | --- |
| **Protozoa**  *Tetrahymena thermophila* | not found | not found | not investigated | not investigated | not investigated | increased by PACAP-38 | chemorepulsion by human PACAP-38 | receptor activation | not investigated | ([Cardoso et al. 2020](#_ENREF_4); [Hassenzahl et al. 2001](#_ENREF_5); [Keedy et al. 2003](#_ENREF_7)) |
| **Porifera** | not found | not found | not investigated | not investigated | not investigated | not investigated | not investigated | not investigated | not investigated | ([Cardoso et al. 2020](#_ENREF_4)) |
| **Placozoa** | not found | not found | not investigated | not investigated | not investigated | not investigated | not investigated | not investigated | not investigated | ([Cardoso et al. 2020](#_ENREF_4)) |
| **Cnetophora** | not found | not found | not investigated | not investigated | not investigated | not investigated | not investigated | not investigated | not investigated | ([Cardoso et al. 2020](#_ENREF_4)) |
| **Cnidaria**  *Hydra magnipapillata* | not found | a partial sequence for PACAP-38 but later studies failed to verify it | not investigated | not investigated | not investigated | not investigated | not investigated | not investigated | not investigated | ([Cardoso et al. 2020](#_ENREF_4); [Kiss and Pirger 2013](#_ENREF_9); [Pirger et al. 2016](#_ENREF_24)) |
| **Planaria**  *Dugesia japonica* | not found | a partial sequence for PACAP-38 but later studies failed to verify it | not investigated | not investigated | not investigated | not investigated | not investigated | not investigated | not investigated | ([Cardoso et al. 2020](#_ENREF_4); [Kiss and Pirger 2013](#_ENREF_9); [Pirger et al. 2016](#_ENREF_24)) |
| **Annelida**  *Lumbricus terrestris* | not  investigated | not found | not investigated | positive signal with mammalian antibodies for PACAP-27 and PACAP-38 | not investigated | not investigated | not investigated | not investigated | not investigated | ([Molnar et al. 2006](#_ENREF_16); [Reglodi et al. 2000](#_ENREF_25)) |
| **Annelida**  *Lumbricus*  *polyphemus* | not  investigated | not  investigated | not investigated | positive signal with a mammalian antibody for PACAP-38 | not investigated | not investigated | not investigated | not investigated | not investigated | ([Reglodi et al. 2000](#_ENREF_25)) |
| **Annelida**  *Eisenia fetida* | not found | not found | not investigated | positive signal with mammalian and fish antibodies for PACAP-27, PACAP-38, and PAC_1_ | positive signal with mammalian antibodies for PACAP-38 and PAC_1_ | not investigated | not investigated | not investigated | not investigated | ([Boros et al. 2008](#_ENREF_2); [Boros et al. 2010](#_ENREF_3); [Molnar et al. 2006](#_ENREF_16); [Molnar et al. 2008](#_ENREF_17); [Somogyi et al. 2009](#_ENREF_26); [Varhalmi et al. 2008](#_ENREF_27)) |
| **Cephalopoda**  *Sepioteuthis lessoniana* | not found | a partial sequence for PACAP-38 but later studies failed to verify it | not investigated | not investigated | not investigated | not investigated | not investigated | not investigated | not investigated | ([Cardoso et al. 2020](#_ENREF_4); [Kiss and Pirger 2013](#_ENREF_9); [Pirger et al. 2016](#_ENREF_24)) |
| **Gastropoda**  *Helix pomatia* | not investigated | not investigated | identified PACAP-like peptide fragments in the CNS | positive signal with mammalian antibodies for PACAP-27, PACAP-38, and PAC_1_ | positive signal with mammalian antibodies for PACAP-27, PACAP-38, and PAC_1_ | increased by PACAP-38 in the homogenate of salivary gland | extracellular application of human PACAP-27/38 transiently depolarized or increased postsynaptic  activity of neurons or elicited a long-lasting hyperpolarization; anti-apoptotic effect in the salivary gland; pretreating the flexor muscle in the posterior tentacle with human PACAP-27 enhanced the nerve-evoked contraction (presynaptic effect on the nicotinic neuromuscular contact); pretreating the flexor muscle with human PACAP-27 enhanced the ACh-evoked contraction (postsynaptic effect) | PACAP-6/27 blocked both presynaptic and postsynaptic effects | DMDA (ACh-R antagonist) or H-7 (PKA and PKC blocker) blocked the presynaptic effect; M65 (PAC_1_ antagonist) or H-7 or ChelCl (PKC blocker) blocked the postsynaptic effect | ([Hernadi et al. 2008](#_ENREF_6); [Krajcs et al. 2015](#_ENREF_10); [Pirger et al. 2008](#_ENREF_20)) |
| **Gastropoda**  *Lymnaea stagnalis* | not found  (this study) | not found  (this study) | identified PACAP-like peptide fragments in the CNS | positive signal with mammalian antibodies for PACAP-38 in the CNS and PAC_1_ in the heart (latter: this study) | positive signal with mammalian antibodies for PACAP-38  (this study) | increased by PACAP-38 in the homogenate of CNS | PACAP is necessary and instructive for the formation of associative memory; systematic application of human PACAP-38 reversed age-related memory impairment; systematic application of human PACAP-38 has a neuroprotective function in dopamine-based neurodegeneration developed in the *Lymnaea* parkinsonian model | PACAP-6/38 reduced the cAMP-increasing effect; PACAP-6/38 blocked the formation of associative memory; PACAP-6/38 blocked the memory-boosting effect | actinomycin-D (transcription inhibitor) blocked the memory-boosting effect | ([Maasz et al. 2017](#_ENREF_13); [Pirger et al. 2010a](#_ENREF_21); [Pirger et al. 2010b](#_ENREF_22); [Pirger et al. 2014](#_ENREF_23)) |
| **Insecta**  *Drosophila melanogaster* | not found | not found | not investigated | positive signal with a mammalian antibody | positive signal with a mammalian antibody | not investigated but AC activity increased by PACAP-38 | focal application of human PACAP-38 to the neuromuscular junction region caused an immediate depolarization and a late, large enhancement of K+ current in muscles;  exposure of  larval neuromuscular preparation to human PACAP-38 enhanced the K+ current; exposure of larval muscle to human PACAP-38 modified calcium  ion transport | PACAP-6/38 abolished the calcium transport effect | 2',5'-dideoxyadenosine (AC inhibitor) abolished the effect on calcium transport;  H-89 (PKA inhibitor) abolished the effect on calcium transport | ([Bhattacharya et al. 2004](#_ENREF_1); [Zhong 1995](#_ENREF_28); [Zhong and Pena 1995](#_ENREF_29)) |
| **Insecta** *Periplaneta*  *americana* | not found | a partial sequence for PACAP-38 but later studies failed to verify it | not investigated | not investigated | not investigated | not investigated | not investigated | not investigated | not investigated | ([Cardoso et al. 2020](#_ENREF_4); [Kiss and Pirger 2013](#_ENREF_9); [Pirger et al. 2016](#_ENREF_24)) |
| **Crustacea**  *Eriocheir japonica* | not investigated | a partial sequence for PACAP-38 | not investigated | not investigated | not investigated | not investigated | not investigated | not investigated | not investigated | ([Cardoso et al. 2020](#_ENREF_4); [Kiss and Pirger 2013](#_ENREF_9); [Pirger et al. 2016](#_ENREF_24)) |
| **Crustacea**  *Litopenaeus vannamei* | not investigated | a partial sequence for PACAP-38 | not investigated | not investigated | not investigated | not investigated | shrimp PACAP-38 in the water boosted innate immunity of the animals (increased hemocyte count, superoxide  dismutase activity, lectins and nitric oxide synthase derived metabolites, total protein concentration, growth) | not investigated | not investigated | ([Lugo et al. 2013](#_ENREF_12)) |

**Supplementary Table 3**. Deuterostome (7 vertebrate species and 2 cephalochordate species) PACAP sequences used as queries for the homolog searching in the *L. stagnalis* sequence data

| **Species** | **Peptide** | **NCBI identifier/reference paper** |
| --- | --- | --- |
| *Homo sapiens* | precursor, PACAP-27/38 | AAB21470 |
| *Mus musculus* | precursor, PACAP-27/38 | BAA28355 |
| *Gallus gallus* | precursor, PACAP-27/38 | AAX56089 |
| *Xenopus laevis* | precursor, PACAP-27/38 | AAD56956 |
| *Danio rerio* | precursors, PACAP-27/38s | NP_690841.1; NP_999880.1 |
| *Oncorhynchus mykiss* | precursor, PACAP-27/38 | AAK28558.1 |
| *Lethenteron japonicum* | precursor, PACAP-27/38 | Ng et al., 2012 ([Ng et al. 2012](#_ENREF_18)) |
| *Branchiostoma floridae* | precursors, PACAP/GCGs | ([Mirabeau and Joly 2013](#_ENREF_15))  ([On et al. 2022](#_ENREF_19)) |
| *Branchiostoma belcheri* | precursors, PACAP/GCGs | ([On et al. 2022](#_ENREF_19)) |

**Supplementary Table 4**. Deuterostome (2 vertebrate species and 1 cephalochordate species) PACAP and PACAP/GCG receptor sequences used as queries for the homolog searching in the *L. stagnalis* sequence data

| **Species** | **Receptor** | **NCBI identifier** |
| --- | --- | --- |
| *Homo sapiens* | PAC_1_ | BAA04466 |
| *Homo sapiens* | VPAC_1_ | NP_004615.2 |
| *Homo sapiens* | VPAC_2_ | NP_003373.2 |
| *Mus musculus* | PAC_1_ | NP_031433.3 |
| *Mus musculus* | VPAC_1_ | NP_035833.2 |
| *Mus musculus* | VPAC_2_ | NP_033537 |
| *Branchiostoma floridae* | bf95 | AKC34087 |

**Supplementary Table 5**. Human Secretin neuropeptide superfamily members as queries for the homolog searching in the *L. stagnalis* sequence data. No direct sequence homologs were found.

| **Query human sequence** | **NCBI identifier** | **Results in the *L. stagnalis***  **sequence data** |
| --- | --- | --- |
| VIP/PHI precursor, active peptides | NP_003372 | no hit |
| PRP | AAB21470 | no hit |
| GHRH | AAB37758 | no hit |
| Secretin | AAG31443 | no hit |
| Preproglucagon precursor,  active peptides | KAI4036670 | no hit |

**Supplementary Figure 1**. Schematic representation of *L. stagnalis* anatomy showing the position of the heart (consisting of an auricle [A] and ventricle [V]) next to the kidney (K) – modified after ([Kerkhoven et al. 1991](#_ENREF_8)) (**a**). Dissected heart from *L. stagnalis* showing the auricle and ventricle. Scale bar = 2 mm (**b**). Abbreviations: LN – lip nerves; CNS – central nervous system; BM – buccal mass; LN – lip nerve; LPN and RPN – left and right parietal nerves; IN – intestinal nerve; AA – anterior aorta; PA – posterior aorta

**
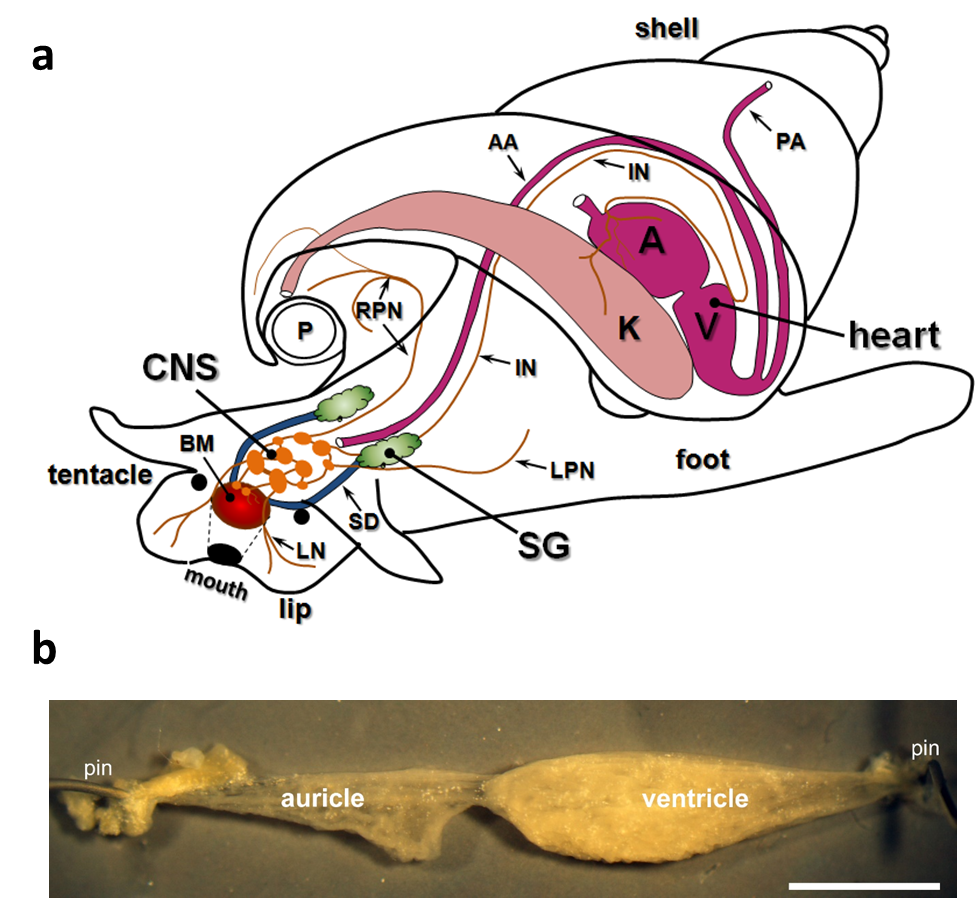
**

**Supplementary Figure 2**. MS/MS spectra of the two main characteristic peptide fragments of synthetic PACAP-38 as the positive control in the immunoprecipitation experiment. (**a**) HSDGIFTDSYSR (1383.62 m/z, [M]^2+^) and (**b**) YLAAVLGK (834.508 m/z, [M + H]^+^).


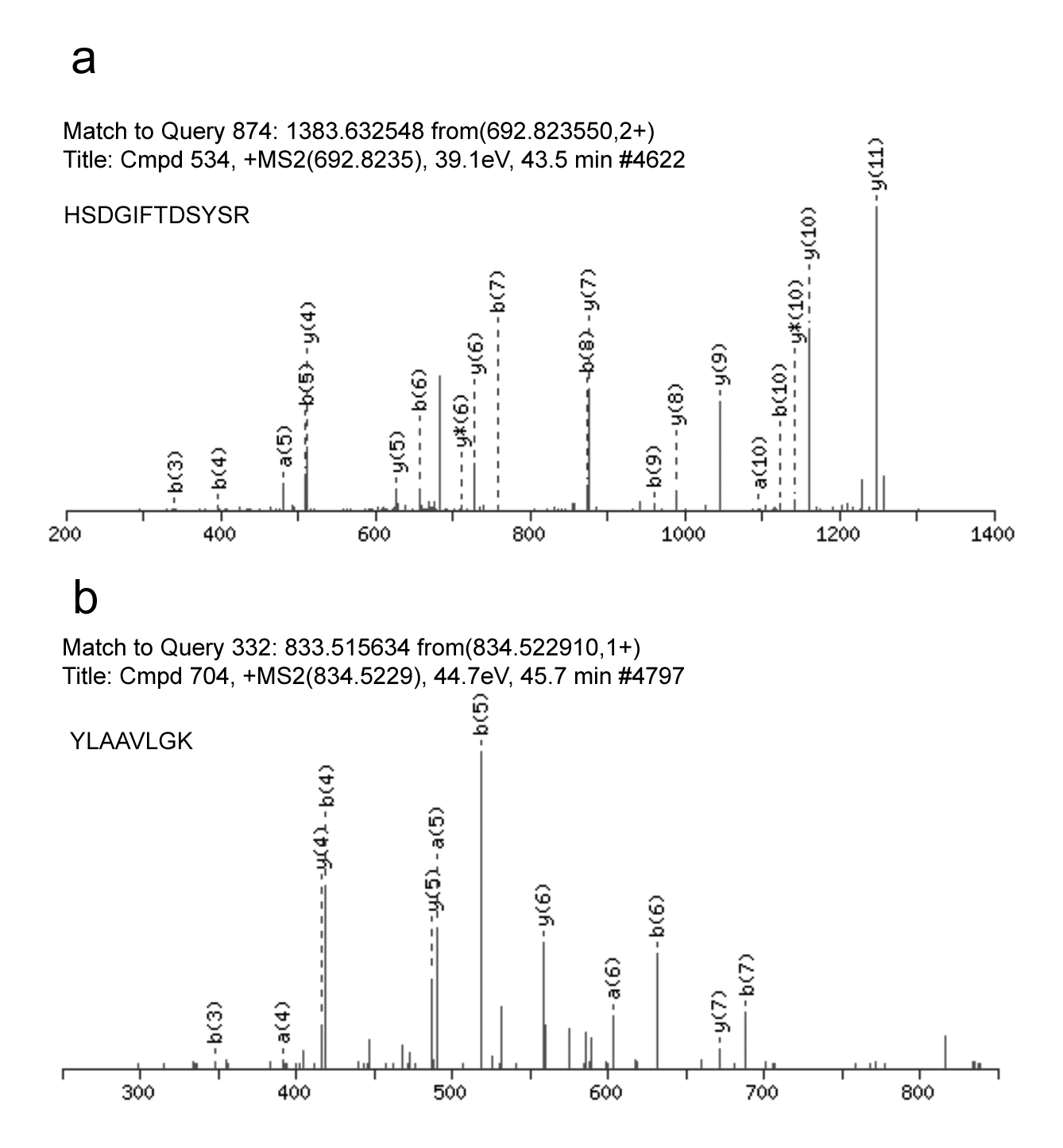


**Supplementary Figure 3**. A representative part of the MS spectrum obtained from the homogenate of the CNS. None of the detected peptide fragments corresponded to the peptide fragments of vertebrate PACAP-38.


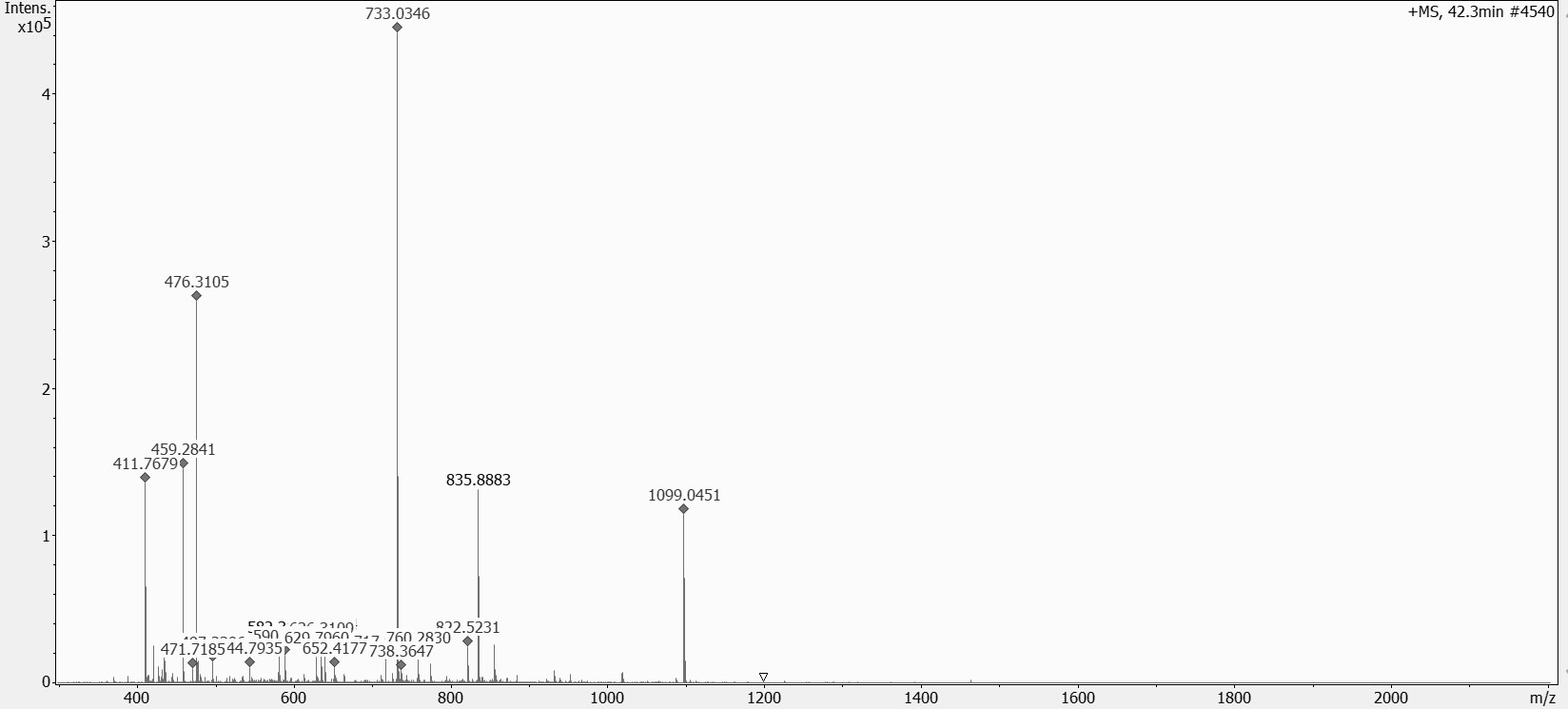


**Supplementary Figure 4.** mRNA (cds) and protein sequence of the identified Cluster B receptor subfamily candidates in *L. stagnalis*. (values with human PAC_1_: candidate 1 – score: 206, E-value: 2e-57; candidate 2 – score: 204, E-value: 1e-57)

>*L. stagnalis* Cluster B receptor candidate 1_mRNA (GenBank accession number: #OR499102)

ATGGGAACGTTACGGAACGACCCCACAAATGCCGTGGACACGTCGAGGAATGTGGTGGTCATTAGTGTGGAGCAGCAACATCAGCTCCTGGAAGAGGCGAGGGTACAGTGTTTGGCGACAATGCTACGGACGAGTCAAGAAGGCTCCGGGCTGAAAGGAAACCACTGCAACATGACCTGGGACGGGATCGTCTGTTGGCCGGCCACACCAGCGGGAAAAACGGTGGAGTTACCTTGCCCCACTTATGTTCATAACTTTTACCTGGGAGCCAAGGCAACGAAAAAATGTATGGACAACGGTGAGTGGTTCTATCGTTCTGAGCTGAACTCCACTTGGACCAACTACACCAGCTGTAGAATGACTGAGGTGTTTGAACACATGGTCCCGCCGCATATTGAATACAAGGATCACATTGAACGAATCAAGATCATGTACAGTGTGGGATACGGCATCTCATTGATAGCGCTGCTCATCGCCATTTTTATTATGATTTATTTTAGACGTCTCCACTGTCCCAGAAACACAATACACCTGAACCTATTCCTGGCCTTCGTCCTACGGGCGCTCCTGTCATTCCTCAAGGACAGTCTGCTGGTGATTCACTTGGGCCTGCGAAATGACGTCATAGAGGTGTCTCCCGGTCAATTCATTTTCGATCCGACAGGATCGCACTGGCAGTGTAAGATGTTGTTCACCACCTTCAACTACGTCCAGCTGACCAGCAGCACGTGGGTCTTCCTGGAAGGTCTTTACTTGTACATTCTCGTCACAGTCACCATCTTCTCAGAACGGCGCTACATTCGCTGGTGTACCATCCTGGGATGGACGGGGCCTCTGCTATTCCTAGTGCCTTGGGTTGTCATTCGTGCCACAGTGGAGGATGAATTGTGTTGGAACACACACCCTACACAAGCTTACTTCTGGATTCTCAAGGGTCCTCAAGTTGCTATGTCAGTTGTCAACTTTATTTTCTTCATCAACATCGTCCGTGTCCTCTTCACCAAATTAGTGCGCACCGCGCCGCCCAGAGCCCGGAAATATAGATACAGACGACTGGGCAAATCTACCCTCGTTTTGATCCCGATCTTCGGTGTCCACTACCTGGTGACCATAGGTGTACCTGACAACATCAACACGGTCATGGAGACCATTAAACTTTACTTTGAAATGTTCTTCAATTCCTTTCAGGGCCTGCTGATAGCGTGTCTGTTCTGCTTCATGAACGGGGAGGTACGAGGAACCGTACAAACTGAAATAAGAAAACGCTACATTAGGCACAAGTTGCGGGTAAATTCCCGAAAGTTCCAGAACAAGTACGTGGCCACGGCCAGCTCACAGGTGCGTCCCAGGACGTCGCCATCTTGTTCCCCGAGGCACGCACGCGCTTGTCCCTCGTGCTCCCCAAGGCACGAGTCCAGAGAGCTGAACTCGTCCAGTGGATCTGATCACACCCTGAGCCTGGAGAGACAGAAGTTGAGGATCTACATCCAGCCCATGCGCTACTACCGCGCCCAACCCCCTATAGCCACGCCCAATGACGCCCGACCCATCATCTACACTAGCAAGCGCAACAGCTACGTCTAA

>*L. stagnalis* Cluster B receptor candidate 1_protein

MGTLRNDPTNAVDTSRNVVVISVEQQHQLLEEARVQCLATMLRTSQEGSGLKGNHCNMTWDGIVCWPATPAGKTVELPCPTYVHNFYLGAKATKKCMDNGEWFYRSELNSTWTNYTSCRMTEVFEHMVPPHIEYKDHIERIKIMYSVGYGISLIALLIAIFIMIYFRRLHCPRNTIHLNLFLAFVLRALLSFLKDSLLVIHLGLRNDVIEVSPGQFIFDPTGSHWQCKMLFTTFNYVQLTSSTWVFLEGLYLYILVTVTIFSERRYIRWCTILGWTGPLLFLVPWVVIRATVEDELCWNTHPTQAYFWILKGPQVAMSVVNFIFFINIVRVLFTKLVRTAPPRARKYRYRRLGKSTLVLIPIFGVHYLVTIGVPDNINTVMETIKLYFEMFFNSFQGLLIACLFCFMNGEVRGTVQTEIRKRYIRHKLRVNSRKFQNKYVATASSQVRPRTSPSCSPRHARACPSCSPRHESRELNSSSGSDHTLSLERQKLRIYIQPMRYYRAQPPIATPNDARPIIYTSKRNSYV

>*L. stagnalis* Cluster B receptor candidate 2_mRNA (GenBank accession number: #OR499103)

ATGATGATAGTAACTGATTGGCGTCAATCGCTATTTAAACAATCTTCAAACTATAATATTTTAAAAAATGGATGGATTGTCATTTTGATCACCCTAACAACCATGATGGAAACTAGCGGGAAACGGCAGCACGGTAGAGCTGTCGTCAACATATCTGTTGATGAACAGGTGGCTCGAATAACGACAGAGCGTGCTCGGTGTCTTGCAACTTATCTTTCAGACTCGAATCTAACAATCAACTCTCAAAACTGTCCAGCTGTCTGGGATGACCTTCTTTGCTGGCCGGAAACCCCTCCTAACACTGTAGCTGTACAGGCTTGTCCTAATTACGTTGAACTGTTCAAACCTTGGGAAAATGCTAGCAGGCAATGCTTGGAGAACGGTTCTTGGTTTTTTGACAGTGTCAATAACCGTACTTGGACAGATCTAACCGCTTGTTTAGATAAAGATTACAGTGATCCACCAGGACAATCACCTCATTTCATCAAGTCTCACATGGGCAGAATTCAACTTATGTACAACGTTGGCTACGGCTTATCATTAGCTTCACTTGTGCTGTCAATATGCATCATGGTCGGCTTCAAAAAGTTGCACTGCTCAAGAAACACCATTCATATGAATCTCTTCCTTTCATTCATCTTGAGAGCTTCATTTTCATTTATGAAGGAAAACTTATTGGTAGAAGGATTGGGATTTCCTTCAGATGTTATTCAAACTGGAGTCGGTGGTGGTGTTATATTTAAAACTGGAATCATGCACTGGGAATGCAAGTTGTTTTTTACATTGTTTCACTACATTCTTGGCGCAAACTACATGTGGATTTTTGCTGAAGCACTCTACTTACATATGATCATTTCAGTAGCAGTTTTCTCGGATAGAGGAAGTACAAAGTGGTACATTCTACTGGGATGGGTTTCACCAGTGGTATTTGTTGTGCCTTGGATCATTGTAAGAGCCACAATGGAAAACATATACTGTTGGAACACACATCCAACACCAGGCTACTTTTGGATTATGAGAGGTCCCATTGTTTTTTCAATCGTGGTCAACTTTGTCTTTTTCCTCAACATAGTGCGGGTCTTGTTCACAAAACTAAACGCTGTGAATTCCCCCGAAGCTAAGAAGTTCAGGTACAGAAAACTAGCCAAATCCACTCTAGTTTTGATTCCATTGTTTGGAATTCACTACATTATATTTGCTGGATTACCAAAAGATGTCAATCAAATGGCTGAGCTCATTCAACTGTATTTTGAAATGTTTTTTAATTCAGTTCAGGGCTTCTTTGTGGCTATCTTGTTCTGCTTTATGAATGGAGAGGTGCAGAGTGAAATAAAAAAGAAGTGGCAGAGGTTTCGCATTACCAGATTCCACCATCTTCCTCAAGGAAGAAATAATAGTCACAATTTGACTTACTCCACATATTTGTCTCGACAACGAGAGTCAAATGCTTCGGTGACTCAAATACAAGAAGCCGGTGACAATCTTGTAAACAATGGGTTTAAAAGACAACATTCAAGTTTGAAACCGAACGAGTTTTCAATGTCCCTACTAACCCAAAAACTCGGATCCCAGGGGCTTCGTGAAAATTCAACGTATTTATTTGGAGACCACGAAAAAATGACTCGAGAAACAAGCTTGAATCAAGACGAAAGCCAGACTCTTTATAATGCTGTTACGTGA

>*L. stagnalis* Cluster B receptor candidate 2_protein

MMIVTDWRQSLFKQSSNYNILKNGWIVILITLTTMMETSGKRQHGRAVVNISVDEQVARITTERARCLATYLSDSNLTINSQNCPAVWDDLLCWPETPPNTVAVQACPNYVELFKPWENASRQCLENGSWFFDSVNNRTWTDLTACLDKDYSDPPGQSPHFIKSHMGRIQLMYNVGYGLSLASLVLSICIMVGFKKLHCSRNTIHMNLFLSFILRASFSFMKENLLVEGLGFPSDVIQTGVGGGVIFKTGIMHWECKLFFTLFHYILGANYMWIFAEALYLHMIISVAVFSDRGSTKWYILLGWVSPVVFVVPWIIVRATMENIYCWNTHPTPGYFWIMRGPIVFSIVVNFVFFLNIVRVLFTKLNAVNSPEAKKFRYRKLAKSTLVLIPLFGIHYIIFAGLPKDVNQMAELIQLYFEMFFNSVQGFFVAILFCFMNGEVQSEIKKKWQRFRITRFHHLPQGRNNSHNLTYSTYLSRQRESNASVTQIQEAGDNLVNNGFKRQHSSLKPNEFSMSLLTQKLGSQGLRENSTYLFGDHEKMTRETSLNQDESQTLYNAVT

**Supplementary Table 6**. Sequence comparison of *L. stagnalis* Cluster B receptors with known protostome and deuterostome B1 GPCRs

| **B1 GPCR sequence** | **Identity and similarity with**  ***L. stagnalis* Cluster B receptor 1** | **Identity and similarity with**  ***L. stagnalis* Cluster B receptor 2** |
| --- | --- | --- |
| *Homo sapiens* PAC_1_ | 25.2 % and 39.8 % | 25.2 % and 39.8 % |
| *Homo sapiens* VPAC_1_ | 29.6 % and 42.1 % | 29.4 % and 43.9 % |
| *Homo sapiens* VPAC_2_ | 28.0 % and 43.6 % | 26.7 % and 40.2 % |
| *Branchiostoma floridae* bf95 | 30.3 % and 49.0 % | 30.1 % and 45.5 % |
| *Daphnia pulex* Cluster B receptor  (DAPPUDRAFT_299719) | 30.9 % and 46.7 % | 29.2 % and 44.8 % |
| *Aplysia californica* Cluster B receptor (XP_005093470) | 28.2 % and 42.4 % | 30.4 % and 42.4 % |

**Supplementary Figure 5**. Conserved domain analysis of protostome and deuterostome B1 GPCR receptor family members. The analysis was performed with NCBI Conserved Domain Search ([Lu et al. 2020](#_ENREF_11); [Marchler-Bauer et al. 2017](#_ENREF_14)). The *L. stagnalis* sequences are indicated with red color.

*Mus musculus* PAC_1_


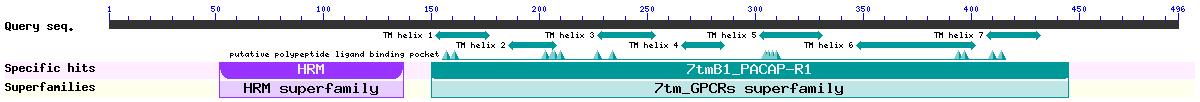


*Mus musculus* VPAC_1_


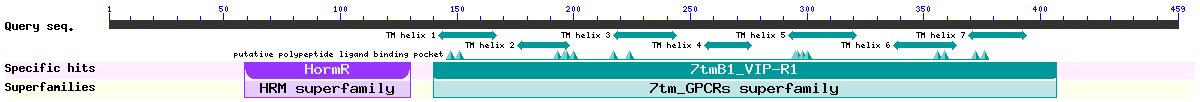


*Mus musculus* VPAC_2_


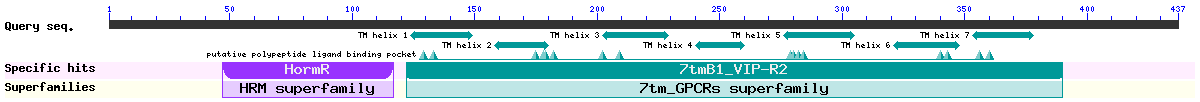


*Branchiostoma floridae* bf95 receptor


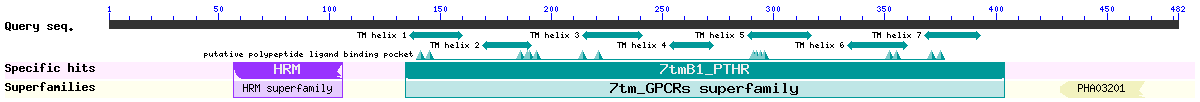


*Daphnia pulex* Cluster B receptor


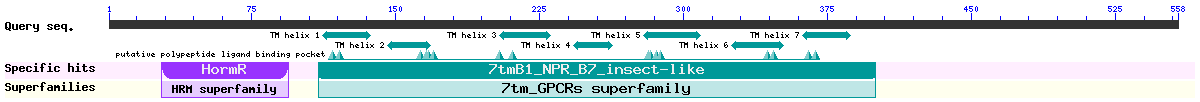


*Aplysia californica* Cluster B receptor


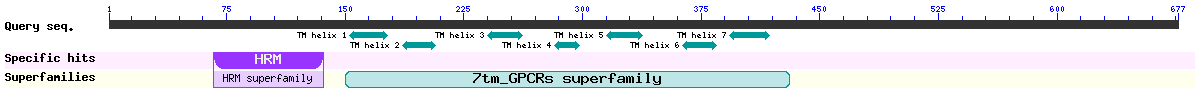


*Lymnaea stagnalis* Cluster B receptor candidate 1


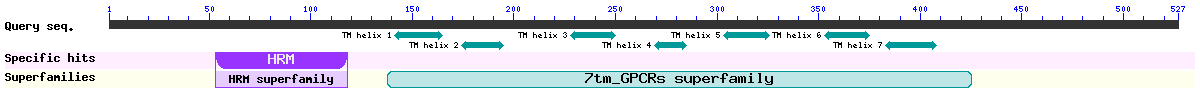


*Lymnaea stagnalis* Cluster B receptor candidate 2


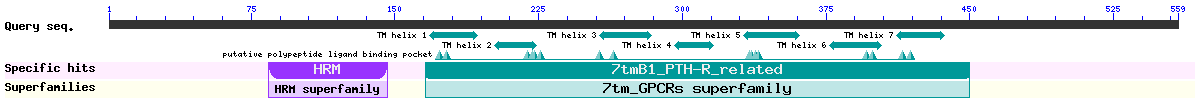


**Supplementary Figure 6**. Generalized sequence conservation of the described invertebrate and vertebrate PACAP mature peptides. Conserved amino acids are indicated with asterisk. If the active peptide sequences were so conserved as they are being supposed, they should be found in the protostome and non-bilaterian genome and transcriptome data.

*:********************************..:*

Human: HSDGIFTDSYSRYRKQMAVKKYLAAVLGKRYKQRVKNK

Rat: HSDGIFTDSYSRYRKQMAVKKYLAAVLGKRYKQRVKNK

Chicken: HIDGIFTDSYSRYRKQMAVKKYLAAVLGKRYKQRVKNK

Frog: HSDGIFTDSYSRYRKQMAVKKYLAAVLGKRYKQRIKNK

Fish: HSDGIFTDSYSRYRKQMAVKKYLAAVLGKRYRQRYRSK

Insect: HSDGIFTDSYSRYRKQMAVKKYLAAVLGKRYRQRYRSK

Mollusk: HSDGIFTDSYSRYRKQMAVKKYLAAVLGKRYRQRYRNK

Cnidaria: HSDGIFTDSYSRYRKQMAVKKYLAAVLGKRYRQRYRNK

**Supplementary Table 7**. *L. stagnalis* proteins with relevant mass to the band detected in the WB as queries for the MS identification

| **Protein** | **Mass (kDa)** | **Results in the *L. stagnalis***  **sequence data** |
| --- | --- | --- |
| CDCH-I | 4.55 | no hit |
| neuropeptide Y | 4.68 | no hit |
| thymosin beta-4 | 4.68 | no hit |
| MIP VII | 4.86 | no hit |
| LYCP III | 5.14 | no hit |
| CDCH-II | 5.44 | no hit |
| HSP40 | 5.44 | no hit |
| metallothionein allelic variant 1 | 5.8 | no hit |
| calcium-binding protein | 5.88 | no hit |

**References**

Bhattacharya A, Lakhman SS, Singh S (2004) Modulation of L-type calcium channels in *Drosophila* via a pituitary adenylyl cyclase-activating polypeptide (PACAP)-mediated pathway. J Biol Chem 279:37291-7. <https://doi.org/10.1074/jbc.M403819200>

Boros A, Reglodi D, Herbert Z, Kiszler G, Nemeth J, Lubics A, Kiss P, Tamas A, Shioda S, Matsuda K et al. (2008) Changes in the expression of PACAP-like compounds during the embryonic development of the earthworm *Eisenia fetida*. J Mol Neurosci 36:157-65. <https://doi.org/10.1007/s12031-008-9102-6>

Boros A, Somogyi I, Engelmann P, Lubics A, Reglodi D, Pollak E, Molnar L (2010) Pituitary adenylate cyclase-activating polypeptide type 1 (PAC1) receptor is expressed during embryonic development of the earthworm. Cell Tissue Res 339:649-53. <https://doi.org/10.1007/s00441-009-0909-4>

Cardoso JCR, Garcia MG, Power DM (2020) Tracing the Origins of the Pituitary Adenylate-Cyclase Activating Polypeptide (PACAP). Front Neurosci 14:366. <https://doi.org/10.3389/fnins.2020.00366>

Hassenzahl DL, Yorgey NK, Keedy MD, Price AR, Hall JA, Myzcka CC, Kuruvilla HG (2001) Chemorepellent signaling through the PACAP/lysozyme receptor is mediated through cAMP and PKC in *Tetrahymena thermophila*. J Comp Physiol A 187:171-6. <https://doi.org/10.1007/s003590100185>

Hernadi L, Pirger Z, Kiss T, Nemeth J, Mark L, Kiss P, Tamas A, Lubics A, Toth G, Shioda S et al. (2008) The presence and distribution of pituitary adenylate cyclase activating polypeptide and its receptor in the snail *Helix pomatia*. Neuroscience 155:387-402. <https://doi.org/10.1016/j.neuroscience.2008.05.003>

Keedy M, Yorgey N, Hilty J, Price A, Hassenzahl D, Kuruvilla H (2003) Pharmacological evidence suggests that the lysozyme/PACAP receptor of *Tetrahymena thermophila* is a polycation receptor. Acta Protozoologica 42:11-17.

Kerkhoven RM, Croll RP, Van Minnen J, Bogerd J, Ramkema MD, Lodder H, Boer HH (1991) Axonal mapping of the giant peptidergic neurons VD1 and RPD2 located in the CNS of the pond snail *Lymnaea stagnalis*, with particular reference to the innervation of the auricle of the heart. Brain Res 565:8-16. <https://doi.org/10.1016/0006-8993(91)91730-o>

Kiss T, Pirger Z (2013) Multifunctional role of PACAP-like peptides in molluscs. Protein Pept Lett 20:628-35. <https://doi.org/10.2174/0929866511320060003>

Krajcs N, Hernadi L, Pirger Z, Reglodi D, Toth G, Kiss T (2015) PACAP Modulates Acetylcholine-Elicited Contractions at Nicotinic Neuromuscular Contacts of the Land Snail. J Mol Neurosci 57:492-500. <https://doi.org/10.1007/s12031-015-0605-7>

Lu S, Wang J, Chitsaz F, Derbyshire MK, Geer RC, Gonzales NR, Gwadz M, Hurwitz DI, Marchler GH, Song JS et al. (2020) CDD/SPARCLE: the conserved domain database in 2020. Nucleic Acids Res 48:D265-D268. <https://doi.org/10.1093/nar/gkz991>

Lugo JM, Carpio Y, Morales R, Rodriguez-Ramos T, Ramos L, Estrada MP (2013) First report of the pituitary adenylate cyclase activating polypeptide (PACAP) in crustaceans: conservation of its functions as growth promoting factor and immunomodulator in the white shrimp *Litopenaeus vannamei*. Fish Shellfish Immunol 35:1788-96. <https://doi.org/10.1016/j.fsi.2013.08.028>

Maasz G, Zrinyi Z, Reglodi D, Petrovics D, Rivnyak A, Kiss T, Jungling A, Tamas A, Pirger Z (2017) Pituitary adenylate cyclase-activating polypeptide (PACAP) has a neuroprotective function in dopamine-based neurodegeneration in rat and snail parkinsonian models. Dis Model Mech 10:127-139. <https://doi.org/10.1242/dmm.027185>

Marchler-Bauer A, Bo Y, Han L, He J, Lanczycki CJ, Lu S, Chitsaz F, Derbyshire MK, Geer RC, Gonzales NR et al. (2017) CDD/SPARCLE: functional classification of proteins via subfamily domain architectures. Nucleic Acids Res 45:D200-D203. <https://doi.org/10.1093/nar/gkw1129>

Mirabeau O, Joly JS (2013) Molecular evolution of peptidergic signaling systems in bilaterians. Proc Natl Acad Sci U S A 110:E2028-37. <https://doi.org/10.1073/pnas.1219956110>

Molnar L, Pollak E, Boros A, Reglodi D, Tamas A, Lengvari I, Arimura A, Lubics A (2006) Comparative anatomy of PACAP-immunoreactive structures in the ventral nerve cord ganglia of lumbricid oligochaetes. Ann N Y Acad Sci 1070:427-30. <https://doi.org/10.1196/annals.1317.056>

Molnar L, Pollak E, Boros A, Shioda S, Nakajo S, Tamas A, Lengvari I, Reglodi D, Lubics A (2008) PAC1 receptor localization in a model nervous system: light and electron microscopic immunocytochemistry on the earthworm ventral nerve cord ganglia. Regul Pept 145:96-104. <https://doi.org/10.1016/j.regpep.2007.09.014>

Ng SY, Chow BK, Kasamatsu J, Kasahara M, Lee LT (2012) Agnathan VIP, PACAP and their receptors: ancestral origins of today's highly diversified forms. PLoS One 7:e44691. <https://doi.org/10.1371/journal.pone.0044691>

On JSW, Su L, Shen H, Arokiaraj AWR, Cardoso JCR, Li G, Chow BKC (2022) PACAP/GCGa Is an Important Modulator of the Amphioxus CNS-Hatschek's Pit Axis, the Homolog of the Vertebrate Hypothalamic-Pituitary Axis in the Basal Chordates. Front Endocrinol 13:850040. <https://doi.org/10.3389/fendo.2022.850040>

Pirger Z, Nemeth J, Hiripi L, Toth G, Kiss P, Lubics A, Tamas A, Hernadi L, Kiss T, Reglodi D (2008) PACAP has anti-apoptotic effect in the salivary gland of an invertebrate species, *Helix pomatia*. J Mol Neurosci 36:105-14. <https://doi.org/10.1007/s12031-008-9070-x>

Pirger Z, Laszlo Z, Kemenes I, Toth G, Reglodi D, Kemenes G (2010a) A homolog of the vertebrate pituitary adenylate cyclase-activating polypeptide is both necessary and instructive for the rapid formation of associative memory in an invertebrate. J Neurosci 30:13766-73. <https://doi.org/10.1523/JNEUROSCI.2577-10.2010>

Pirger Z, Laszlo Z, Hiripi L, Hernadi L, Toth G, Lubics A, Reglodi D, Kemenes G, Mark L (2010b) Pituitary adenylate cyclase activating polypeptide (PACAP) and its receptors are present and biochemically active in the central nervous system of the pond snail *Lymnaea stagnalis*. J Mol Neurosci 42:464-71. <https://doi.org/10.1007/s12031-010-9361-x>

Pirger Z, Naskar S, Laszlo Z, Kemenes G, Reglodi D, Kemenes I (2014) Reversal of Age-Related Learning Deficiency by the Vertebrate PACAP and IGF-1 in a Novel Invertebrate Model of Aging: The Pond Snail (*Lymnaea stagnalis*). J Gerontol A Biol Sci Med Sci 69:1331-1338. <https://doi.org/10.1093/gerona/glu068>

Pirger Z, Krajcs N, Kiss T (2016) Occurrence, Distribution, and Physiological Function of Pituitary Adenylyl Cyclase-Activating Polypeptide in Invertebrate Species. in Pituitary Adenylate Cyclase Activating Polypeptide — PACAP (eds D. Reglodi & A. Tamas), Current Topics in Neurotoxicity Vol. 11 (Springer Nature, New York).

Reglodi D, Lengvari I, Szelier M, Vigh S, Arimura A (2000) Distribution of PACAP-like immunoreactivity in the nervous system of oligochaeta. Peptides 21:183-8. <https://doi.org/10.1016/s0196-9781(99)00201-6>

Somogyi I, Boros A, Engelmann P, Varhalmi E, Nemeth J, Lubics A, Tamas A, Kiss P, Reglodi D, Pollak E et al. (2009) Pituitary adenylate cyclase-activating polypeptide-like compounds could modulate the activity of coelomocytes in the earthworm. Ann N Y Acad Sci 1163:521-3. <https://doi.org/10.1111/j.1749-6632.2009.04431.x>

Varhalmi E, Somogyi I, Kiszler G, Nemeth J, Reglodi D, Lubics A, Kiss P, Tamas A, Pollak E, Molnar L (2008) Expression of PACAP-like compounds during the caudal regeneration of the earthworm *Eisenia fetida*. J Mol Neurosci 36:166-74. <https://doi.org/10.1007/s12031-008-9125-z>

Zhong Y (1995) Mediation of PACAP-like neuropeptide transmission by coactivation of Ras/Raf and cAMP signal transduction pathways in Drosophila. Nature 375:588-92. <https://doi.org/10.1038/375588a0>

Zhong Y, Pena LA (1995) A novel synaptic transmission mediated by a PACAP-like neuropeptide in Drosophila. Neuron 14:527-36. <https://doi.org/10.1016/0896-6273(95)90309-7>
